# High caloric intake improves neuronal metabolism and functional hyperemia in a rat model of early AD pathology

**DOI:** 10.7150/thno.98793

**Published:** 2024-10-28

**Authors:** Dustin Loren V. Almanza, Margaret M. Koletar, Aaron Y. Lai, Wilfred W. Lam, Lewis Joo, Mary E. Hill, Greg J. Stanisz, JoAnne McLaurin, Bojana Stefanovic

**Affiliations:** 1 Physical Sciences Platform, Sunnybrook Research Institute, Toronto, Canada.; 2 Biological Sciences Platform, Sunnybrook Research Institute, Toronto, Canada.; 3 Department of Medical Biophysics, University of Toronto, Toronto, Canada.; 4 Department of Neurosurgery and Pediatric Neurosurgery, Medical University, Lublin, Poland.; 5 Department of Laboratory Medicine and Pathobiology, University of Toronto, Toronto, Canada.

**Keywords:** Alzheimer's disease, TgF344-AD, obesity, diet, glucose metabolism, MRI, CEST, functional hyperemia

## Abstract

**Introduction:** While obesity has been linked to both increased and decreased rate of cognitive decline in Alzheimer's Disease (AD) patients, there is no consensus on the interaction between obesity and AD.

**Methods:** The TgF344-AD rat model was used to investigate the effects of high carbohydrate, high fat (HCHF) diet on brain glucose metabolism and hemodynamics in the presence or absence of AD transgenes, in presymptomatic (6-month-old) vs. symptomatic (12-month-old) stages of AD progression using non-invasive neuroimaging.

**Results:** In presymptomatic AD, HCHF exerted detrimental effects, attenuating both hippocampal glucose uptake and resting perfusion in both non-transgenic and TgAD cohorts, when compared to CHOW-fed cohorts. In contrast, HCHF consumption was beneficial in established AD, resolving the AD-progression associated attenuation in hippocampal glucose uptake and functional hyperemia.

**Discussion:** Whereas HCHF was harmful to the presymptomatic AD brain, it ameliorated deficits in hippocampal metabolism and neurovascular coupling in symptomatic TgAD rats.

## Introduction

Alzheimer's disease (AD) is the most common cause of dementia 1. Worldwide dementia is expected to rise to more than 150 million by 2050 2. The AD brain is histopathologically characterized by abnormal accumulation of beta-amyloid (Aβ) plaques outside neurons, and neurofibrillary tangles (NFTs) inside neurons 3. Currently, there are only two FDA approved anti-Aβ monoclonal antibodies (aducanumab and lecanemab). The clinical benefit, i.e., slower functional and cognitive decline, of aducanumab, the first FDA approved drug to reduce the quantity of Aβ plaques in AD patients 4,5, has not yet been achieved in AD patients 4,6. Lecanemab, despite being associated with some adverse events, shows reduction of amyloid beta plaques with moderately slower decline on measures of cognition, when compared to placebo 7. As the effectiveness of Aβ plaque reduction strategies for abating cognitive decline has been very modest 7-9, AD management is for the most part still restricted to symptom treatment 10 using e.g., acetylcholinesterase inhibitors and non-competitive N-methyl-d-aspartic acid (NMDA) receptor antagonists 11. In addition to amyloid and tau accumulation, brain metabolism changes can serve as a measure of neuropathology, as it is strongly correlated to neuronal functioning 12-14. Brain glucose metabolism is reduced in normal aging and further decreased in AD 15-17, with an accompanied drop in cerebral blood flow 18. Aggravated glucose hypometabolism and cerebral hypoperfusion 19,20 typically arise before the clinical onset of AD 21,22.

Multiple studies have suggested an interaction between AD and obesity 23,24, showing higher AD incidence in the obese population. High calorie diets that induce obesity increase AD risk in middle-aged adults 25. Obesity has been shown to exacerbate Aβ deposition-associated cognitive decline 26, and it is also associated with cerebrovascular dysfunction and disease 27. Obese adults exhibit reduced cerebral blood flow (CBF) 28 and blood brain barrier (BBB) dysfunction 29, potentially leading to increased Aβ production 30, neuronal dysfunction, and ultimately cognitive decline 30. In contrast to adverse effects of midlife obesity on AD, late-life (>70 years old) obesity is associated with reduced AD progression risk 31, although some have argued that this is a manifestation of prodromal AD-associated weight loss 32 since weight loss in mild cognitive impairment (MCI) increases the risk of AD and dementia 33. Late life obesity correlates with about 20% lower risk of cognitive impairment and mortality 34, as well as a slower decline in cognitive function in individuals with MCI 31. This contrast in the effect of excess weight on AD risk between middle-aged vs. older populations, has been termed the "obesity paradox" 35. The objective of the study was to elucidate the effects of high-carbohydrate, high-fat diet on brain glucose metabolism and hemodynamics in the presence of AD transgenes, at different stages of AD pathology progression. We speculated that enhanced metabolic support may be propitious to the metabolically dysregulated AD brain.

Investigating obesity's impact on AD progression in clinical settings is challenging due to recruitment difficulties, longitudinal observations, dietary controls, comorbidities, and other factors causing obesity. Finely controlled longitudinal experiments thus necessitate the use of experimental models. High carbohydrate/fat diet (HCHF) exposure prior to amyloidogenesis accelerates cognitive decline and exacerbates AD-related pathology in several AD animal models, e.g., 6.5-month-old TgF344-AD rats 36 and 4-month-old APP/PS1 mice 37. In contrast, high-fat diet feeding of 3-month-old Tg6799 AD mice before established AD pathology shows protective effects, reducing Aβ deposition and improving cognitive function 38. The current study used the TgF344-AD rat 39 that expresses the Swedish mutant human APP and Δ exon 9 mutant human presenilin-1. This AD model recapitulates key features of AD pathology (Aβ plaques, neurofibrillary pre-tangles, neuronal loss, chronic neuroinflammation, and bioenergetic impairments), and progressive cognitive decline. Up to 6 months of age (presymptomatic AD), TgF344-AD rats from our colony show no significant behavioural compromise 40 and very low amyloid beta 41. At 9 months (early AD), TgF344-AD rats show hippocampal accumulation of amyloid plaques and tau hyperphosphorylation 42,43, cerebrovascular dysfunction, and reduced hippocampal glucose uptake 44. At 12 months (symptomatic AD), our TgF344-AD rats exhibit memory impairments 45, amyloid plaques accumulation, and tau hyperphosphorylation 41,45. Lai *et al.* showed that at 12 months of age, HCHF diet-induced obesity slowed cognitive decline in TgF344-AD rats, specifically their executive function, as measured in the reversal stage of the Barnes' maze, without affecting the non-transgenic rats 46. In addition, pathological analyses of their hippocampus showed that HCHF-induced obesity in TgF344-AD rats increased density of myelin and oligodendrocytes, and lowered density and activation of microglia that may underlie their cognitive improvement 46. Notwithstanding, obesity also decreased neuronal density, and increased deposition of amyloid-beta plaques and tau inclusions 46. At 15 months, TgF344-AD rats display significant deficits in spatial memory and executive function 40. In other colonies of this model, the mitochondrial complex has been shown to be downregulated 47 in the absence of any progressive weight loss. While 6.5-month-old TgF344-AD rats have shown exacerbated disease progression on Western diet 36, AD stage-dependent effects of such dietary interventions have not been tested to date, yet may be key to understanding the obesity paradox. In particular, we speculated that the onset of diet intervention, relative to the AD stage, determines the nature of the diet's effects on AD pathology progression.

To this end, we have characterized the effect of high carbohydrate and high fat (HCHF) diet-induced obesity in presymptomatic AD (at 6 months) *vs.* symptomatic AD (at 12 months) stages on brain glucose metabolism and hemodynamics in TgF344-AD rats. To emulate obesity in the human population more accurately, TgF344-AD rats and their non-transgenic (nTg) littermates were exposed to either CHOW only or a combination of CHOW and a time-varying selection of HCHF food items. The dietary intervention was commenced either at 6 months of age (before the onset of cognitive decline) or at 12 months of age (established stage of the disease). Chemical exchange saturation transfer (CEST) was employed to quantify *in situ* glucose uptake, whereas pseudo continuous arterial spin labeling (pCASL) MRI was used to quantify cerebral blood flow and cerebrovascular reactivity to neuronal stimulation, i.e., the functional hyperemia. Our findings demonstrate that the effects of obesity on AD - but not healthy - brain physiology depend on the stage of its induction. Whereas HCHF diet was detrimental to the nTg rats irrespective of the time of diet administration, HCHF diet was harmful in the presymptomatic, but beneficial in the symptomatic stage of AD in the TgF344-AD animals. We thus provide the first experimental evidence for the underpinnings of the obesity paradox by leveraging noninvasive MRI techniques in an experimental model of AD comorbid with obesity.

## Methods

### Animals

A total of 85 rats were used in this study and were divided into five groups. Nontransgenic and TgF344AD rat colonies at Sunnybrook were given *ad libitum* standard chow alone (CHOW group); or *ad libitum* chow plus high carbohydrate and high fat diet (HCHF group) for three months prior to MRI, with *ad libitum* access to water alone (CHOW group) or water and a 12% w/v sucrose solution (HCHF group). The HCHF group thus received CHOW and water, supplemented with three highly palatable items, and a 12% w/v sucrose solution. To maintain interest and thus continued caloric overconsumption and weight gain, the highly palatable food combinations were alternated, as described previously 48, based on animals' dietary preferences, as determined in pilot experiments. The HCHF diet was administered for three months following earlier work on the evolution of diet effects in rats 48 before examining the rats at 6 months or 12 months of age, corresponding to the presymptomatic and symptomatic AD stages of AD pathology progression in our colony. To investigate whether the observed effects of HCHF diet on the brain metabolism in the symptomatic TgAD rats (at 12 months) would be sustained, a separate cohort of 9-month-old rats were fed with standard CHOW supplemented with HCHF items for 6 months starting from 9 months of age. The feeding paradigm was designed to evaluate the transient induction of obesity through an HCHF diet at different stages of AD pathology progression; rather than focusing on the effects of long-term application of the diet in TgF344-AD rats, which we believe to be detrimental. The summary of animal numbers per experiment is provided in Table 1*.* Experimental protocols were approved by the Animal Care Committee of the Sunnybrook Health Sciences Center, which adheres to the guidelines and policies of the Canadian Council on Animal Care, and meets all the requirements of the Provincial Statute of Ontario, Animals for Research Act and Federal Health of Animals Act. The TgF344-AD rats colony was started following donation of breeders by Dr. Terrence Town; these animals overexpress Swedish mutant human APP (APPswe: APP KM670/671NL) and delta exon 9 mutant of human PS1 (PS1ΔE9) 39. The rats were kept in a reverse light cycle (12-hour dark:light, light from 7pm to 7am) to facilitate glucose uptake measurements in the active phase, which are critical for high fidelity of this assay 49-51.

### Experimental design

Prior to MRI imaging, rats were fasted overnight (~15hrs) corresponding to the rat's inactive phase to enhance glucose uptake contrast 52,53; although stress response was not presently investigated, this duration of fasting has not been previously shown to induce stress in adult rats 54. The rats were then imaged during the dark phase of their light cycle, corresponding to their active period 55. The total imaging time was about five hours. Briefly, the imaging protocol comprised the acquisition of a multislice 2D structural scan, pseudo continuous arterial spin labeling for perfusion and functional hyperemia measurements, and chemical exchange saturation transfer (CEST) for 2DG uptake measurement. At the beginning of the CEST scan, 2DG (at 1g/kg in 0.33 g/ml solution) was injected through the tail vein catheter.

**Figure 1:**
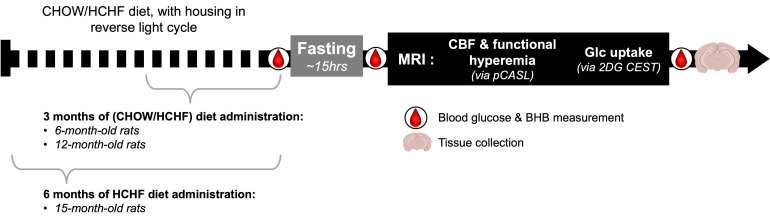
** Study Timeline**. A total of five groups were used in the study. Animals were scanned at 6 months and 12 months of age after feeding them either CHOW alone or CHOW and HCHF food items for 3 months. An additional group was scanned at 15 months of age after these diets were administered for 6 months. Blood glucose and BHB were measured before fasting, after fasting, and at the end of MRI scan. Rats were fasted during their inactive phase, to administer the 2DG uptake in the active phase, thereby enhancing the 2DG uptake and potentiating the MRI contrast (relative to those achievable in the inactive phase).

Weight, blood glucose, and β-Hydroxybutyric acid (BHB) were measured before and after fasting to assess the effects of fasting. To confirm successful delivery of 2DG, blood glucose and BHB were also measured at the end of the experiment (with the blood collected via cardiac puncture). Blood glucose and BHB were measured using glucose and BHB strips with a glucometer (FreeStyle Precision Neo, Abbott, Mississauga, Ontario, Canada). The timeline of the experimental procedures is shown in ***Figure 1***.

### *In vivo* experiments

The rats were anesthetized with isoflurane (5% induction and 1.75-2% for maintenance). Subsequently, 0.1 ml of blood was drawn from the tail vein for post-fast blood glucose and BHB measurements before tail vein cannulation for injection of 2-deoxy-glucose (2DG; 1 g/kg in a 0.33 g/ml mixture; Santa Cruz Biotechnology, Dallas, TX, USA). Rats were ventilated with 30% O_2_ and 70% N_2_ controlled by a programmable gas mixer (GSM-3, CWE Inc., Ardmore, PA, USA), and transferred to the MRI bed. The systemic state was monitored throughout the session using a pulse oximeter (MouseOx Plus, Starr Life Sciences Corp., Oakmont, PA, USA), pneumatic pillow under the abdomen, and a rectal temperature probe (Model 1025, SA Instruments Inc., Stony Brook, NY, USA). Body temperature was maintained at 37 ± 1°C using a water-circulating heating pad (SA Instruments Inc., Stony Brook, NY, USA).

Rats were scanned in a 7T horizontal bore MRI scanner (BioSpec 70/30 USR with BGA-12SHP gradients running ParaVision 6.0.1, Bruker BioSpin, Billerica, MA, USA). A 20-mm diameter loop receive coil was placed over the animal's head and an 86-mm diameter volume coil was used for excitation. Ear and incisor bars were used to secure the rat's head and positioned inside the magnet such that the center of the brain volume of interest was at the isocenter. Structural scan comprised a multislice 2D T2-weighted rapid acquisition with refocused echoes (RARE) sequence (TR/TE_eff_ = 2500/ 44 ms, RARE factor = 8, FOV (field of view) = 20 × 20 mm^2^, in-plane resolution = 0.2 × 0.2 mm^2^, slice thickness = 1 mm, averages = 2). ParaVision's Map Shim was run to minimize B_0_ inhomogeneities.

### 2DG CEST acquisition and analysis

The saturation transfer-weighted FLASH sequence utilized a 490-ms radiofrequency saturation pulse with an amplitude of 1.5 μT per k-space line, followed by a single-slice gradient echo recalled acquisition. The acquisition parameters were as follows: TR/TE = 500/3 ms, flip angle = 30°, field of view (FOV) = 20 × 20 mm^2^, in-plane resolution = 0.31 × 0.31 mm^2^, and slice thickness = 1.5 mm 56. The cumulative saturation time during the center of k-space acquisition was approximately 16 s. The uptake of 2DG was estimated by probing the CEST signal at specific frequency offsets (2.0 ppm and 2.9 ppm for the -OH group at the first carbon and 1.2 ppm for the -OH groups at the third and fourth carbon of the 2DG molecule). The uptake of 2DG was assessed by dynamic CEST imaging, and the signal decreases at 1.2 ppm, 2.0 ppm, and 2.9 ppm were taken to reflect the changes in the concentration of 2DG hydroxyl groups 44,52,53,57,58. The offset farthest from the water resonance, at 2.9 ppm, was reported to sufficiently measure and show 2DG uptake. Baseline data were acquired over 20 mins with 12 repetitions of each offset, followed by 2DG injection and another 60 minutes of post-2DG data acquisition. For each offset, a total of 48 data points were collected from baseline up to 60 minutes post-2DG administration. The dynamic acquisition comprised a dummy scan, a reference scan at a frequency offset of 667 ppm (to avoid saturation transfer effects while providing a baseline measurement to correct for factors such as B_0_ field inhomogeneity, signal drift and a reference point for normalizing the CEST signal acquired), 12 repetitions of a three-offset acquisition at 1.2 ppm, 2.0 ppm, and 2.9 ppm, and another reference scan at a frequency offset of 667 ppm at the end of the acquisition to account for signal drift caused by experimental conditions or the system over the course of the dynamic acquisition 44,53,59,60. This 20.7-minute segment was acquired four times, with the first segment acquired during baseline and the subsequent three segments spanning the one-hour period after the onset of the 2DG injection.

A line was fitted to the initial and final reference scans for each of the four segments voxel-wise and interpolated to the measured time points. Scanner baseline drift correction and signal normalization were done by dividing the time series for each segment by the interpolated reference measurements. The magnetization transfer ratio (MTR; defined as *1 - S/S_0_*) was calculated where *S* is the reduced water signal by the radiofrequency saturation pulse (with amplitude B_1_) at the frequencies of exchanging molecules while *S_0_* is the signal without saturation. The CEST time course data was normalized based on the average signal magnitude of the baseline (first 20 mins of the CEST imaging protocol) before 2DG injection and reported as the difference in magnetization transfer ratio from baseline, i.e., ΔMTR = MTR - MTR_baseline_. The CEST data normalization was performed so as to generate the MTR change (ΔMTR) time courses 58,61. To quantify the uptake of glucose, the area under the curve (AUC) from the onset of 2DG injection up to 60 mins after 2DG injection onset was evaluated (*numpy.trapz* in Python) 62. The AUC of each segment was also evaluated to coarsely assess glucose uptake phases: initial phase representing early responses, peak phase containing maximum uptake, and washout phase corresponding to the decline/stabilization of uptake.

### Perfusion acquisition and analysis

The pseudo continuous arterial spin labeling (pCASL) acquisition protocol and analysis followed our previous work 44. Unbalanced labeling with 5000 RF pulses was employed, each lasting 400-µs with 4 µT, B_1_ amplitude, G_max_/G_mean_ of 45/10 mT/m and 800 µs inter-pulse interval, with the inter-pulse phases optimized to maximize ASL contrast 63. The imaging slice was positioned axially at -0.4 mm AP, maximizing the cross-sectional area of the forelimb region of primary somatosensory cortex (S1FL); the labeling plane was placed 19 mm caudal from the imaging plane. To measure the relative perfusion change in response to bilateral electric forepaw stimulation, a single post labeling delay (PLD) pCASL was used 64 with ***τ*** = 2 seconds (labeling duration), and w = 0.35 s (post labeling delay). We acquired 264 repetitions of single-slice label-control EPI pairs (TR/TE = 2500/11 ms, 0.32 mm in-plane resolution, 1 mm slice thickness) during 4 mins of baseline alternating with three 2-min periods of bilateral forepaw electric stimulation (10 mA, 0.3 ms pulses delivered at 3 Hz). The 4-min baseline acquisition was used to estimate resting cerebral blood flow (CBF) using the model as described previously 65,66, with ***λ*** = 0.9 mL/g (blood-brain water partition coefficient) 67, R_1a_ = 0.45 s^-1^ (longitudinal relaxation time of blood) 68, and ***α*** = 0.85 (labeling efficiency based on preliminary phantom experiments).

The pCASL images were analyzed as described previously 44,69. At first, the pCASL images were masked (Aedes, Matlab), motion corrected (*2dImReg*, AFNI, Analysis of Functional NeuroImages), and low pass filtered (*3dBlurToFWHM*, AFNI; 0.55 mm FWHM). The pCASL data were then fit to a generalized linear model (*3dDeconvolve*, AFNI) producing least squares estimates of the linear regression coefficients with corresponding statistics, thresholding at a false discovery rate of q < 0.05 and minimum cluster size of 4 44.

### Immunofluorescence and immunohistochemistry

Upon completion to diet exposure and/or MRI imaging, rats were transcardially perfused under 5% isoflurane with PBS-0.1% heparin followed by PBS-4% paraformaldehyde before brains were extracted, post-fixed overnight in PBS-4% paraformaldehyde, and cryopreserved in PBS-30% sucrose for microtome sectioning. For each brain, three 40-µm sections evenly spaced between -3.0 mm AP and -4.5 mm AP were sampled.

For immunofluorescence, sections analyzed for cerebral amyloid angiopathy (CAA) were first incubated with 1% Thioflavin S (Sigma #T1892) in water for 8 minutes, then washed twice in 70% ethanol for 3 minutes each before blocking. All sections were blocked in 0.5% Triton X-100 and 0.5% bovine serum albumin in PBS for 1 hour, then probed overnight with the following primary antibodies/dyes diluted in block: Anti-aquaporin 4 (AQP4, 1:500, Millipore #AB3594), tomato lectin-Texas Red (1:250, Vector #TL-1176-1) followed by 2 hour labeling with the appropriate secondary antibodies diluted in block: Anti-rabbit IgG-Alexa 488 (1:250, ThermoFisher #A21206), anti-rabbit IgG-Alexa 594 (1:250, ThermoFisher #A11037), before mounting.

Zeiss Observer.Z1. was used to acquire fluorescence images, which were analyzed using Fiji. For analysis of CAA, 25-30 vessels > 15 µm in diameter were included per rat. Each vessel was assigned a CAA score based on fluorescence coverage of Thioflavin S (ThioS) on lectin, using previously established criteria 70. For analysis of AQP4 immunoreactivity, three tiled images of the whole hippocampus were included per rat.

### Statistical analysis

Tests for normality of the data distribution in each contrast were performed using the Shapiro-Wilk test implemented in the *scipy.stats* module in Python (Version 1.7.3.) 71. A linear mixed-effects model was used, which included effects of age, diet, genotype, and sex, and their interactions with blood glucose, brain glucose uptake, resting CBF, extent of the brain showing significant CBF changes in response to forepaw stimulation, and the magnitude of CBF responses to forepaw stimulation. The model was implemented in the *MixedLM* class from the *statsmodel* library in Python. The mixed-effects model employed was used to analyze relationships between dependent variable (e.g., brain glucose uptake) and a combination of independent variables (e.g., age, diet, genotype, sex) and interactions between these independent variables. For example: *brain_glucose_uptake = β_0_diet + β_1_genotype + β_2_sex + β_3_rat_id + ϵ* where *β_0_*,* β_1_*, *β_2_* are the fixed effect coefficients that represents the average change in response variable (b*rain_glucose_uptake*) associated with one-unit change in corresponding predictor (e.g., diet, genotype) while holding other variables constant; *β_3_* is a random effect coefficient; *rat_id* is the random effect grouping variable, and *ϵ* is the error term. This formula specifies the fixed predictors (age, diet, genotype, sex) of the independent variable (e.g., area under the curve in the CEST time course obtained at 2.9ppm reflecting the brain glucose uptake) and incorporated the random effect of the grouping variable “*rat_id*” allowing the model to estimate variability between rats. Subsequently, pairwise comparisons using Tukey's Honestly Significant Difference (HSD) test via the *statsmodels.stats.multicomp.pairwise_tukeyhsd* function (Version 0.13.5) in Python 72 for normally distributed data sets; and Mann-Whitney U test using *scipy.stats* module in Python (Version 1.7.3.) 71 for not normally distributed data sets.

## Results

### Weight change after HCHF consumption

**Figure 2:**
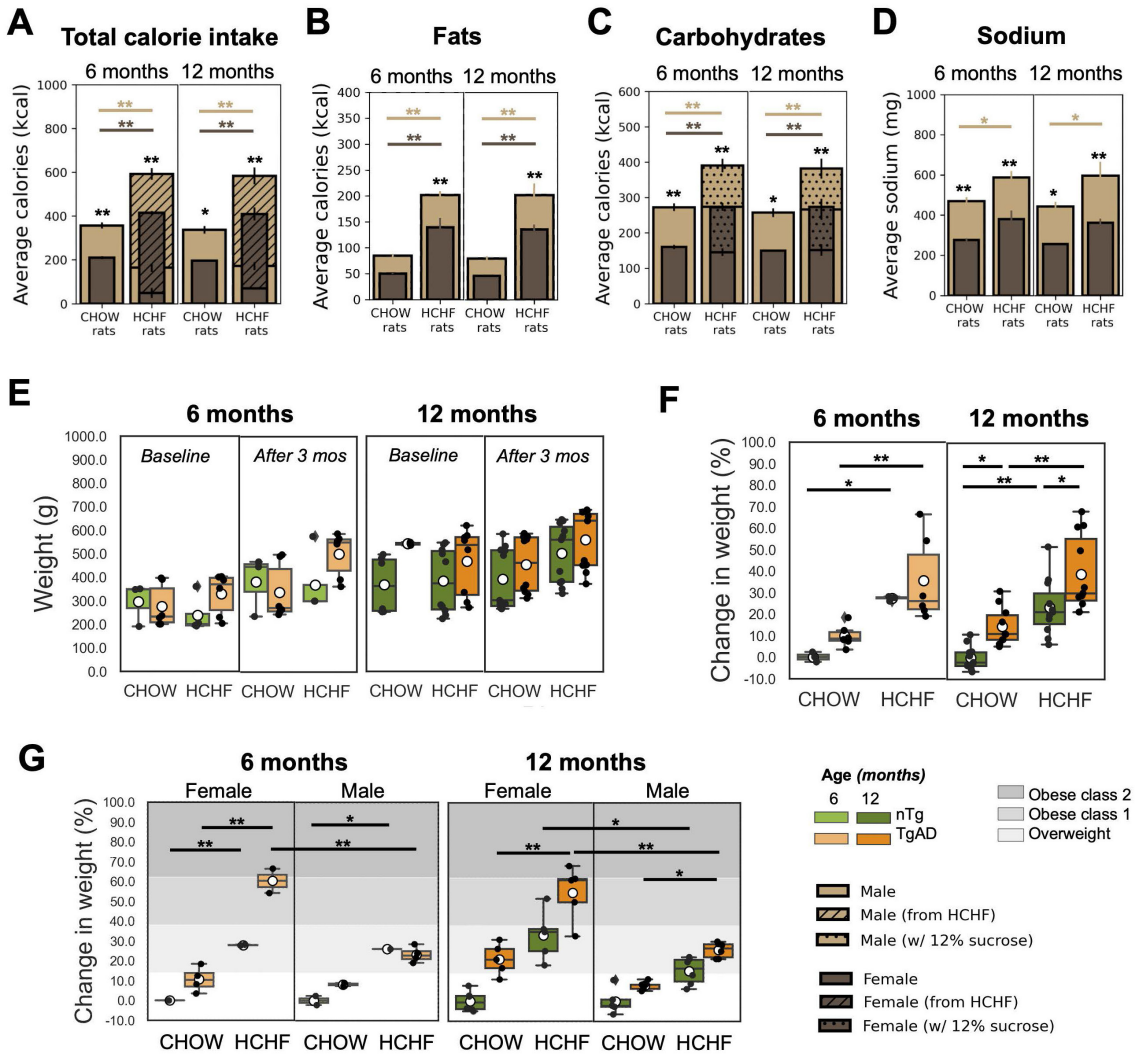
** HCHF elicits weight gain, particularly in females. (A)** Caloric intake was higher in HCHF-fed rats than in their CHOW counterparts (6 months: 206 ± 17 kcal/week, *P < 0*.001; 12 months: 213 ± 33 kcal/week, *P < 0*.001). HCHF-fed rats consumed lower CHOW food compared to CHOW-fed rats (6 months: -165 ± 32 kcal/week, *P = 0*.009; 12 months: -169 ± 29 kcal/week, *P = 0*.004). Compared to CHOW-fed rats, HCHF-fed rats consumed higher **(B)** fats (6 months: 127 ± 29 kcal/week, *P = 0*.033; 12 months: 111 ± 27 kcal/week, *P = 0*.043), and **(C)** carbohydrates (6 months: 165 ± 32 kcal/week, *P = 0*.009; 12 months: 169 ± 29 kcal/week, *P = 0*.004). However, the total carbohydrates consumed by HCHF-fed rats was comparable to CHOW-fed rats without the consumption of 12% sucrose. **(D)** Sodium intake of HCHF-fed male rats was higher than CHOW-fed male rats (6 months: 117 ± 19 mg/week, *P = 0*.027; 12 months: 155 ± 26 mg/week, *P = 0*.011). **(E)** Weight at baseline and after 3 months of either CHOW or HCHF diet. **(F)** HCHF-fed rats gained more weight compared to their CHOW-fed littermates (6 months: 27 ± 7% higher weight gain, *P < 0*.001; 12 months: 23 ± 5% higher weight gain, *P < 0*.001). At 12 months, TgAD rats gained more weight than nTg rats (CHOW-fed rats: 15 ± 4%, *P = 0*.047; HCHF-fed rats: 16 ± 4%, *P = 0*.025). The average weight of CHOW-fed nTg cohorts was considered as the normal weight for each age and used as the reference in computing the weight elevation for each rat.** (G)** The gray-shaded regions indicate the weight gain level classified as overweight [14%, 38%], obese class 1 [38%, 62%], or obese class 2 [62%, 84%], as defined from human BMI ranges 73. At 12 months of age, HCHF-fed female rats had higher increase in weight (by 15 ± 4% in nTg, *P = 0*.016; by 29 ± 4% higher weight gain in TgAD, *P < 0*.001) compared to HCHF-fed male rats. At 6 months of age, n=20, where n=9 [nTg(1 female, 2 male); TgAD(4 female, 2 male)] were CHOW-fed and n=11 [nTg(3 female, 2 male); TgAD(2 female, 4 male)] were HCHF-fed. At 12 months of age, n=43, where n=21 [nTg(6 female, 5 male); TgAD(5 female, 5 male)] were CHOW-fed and n=22 [nTg(5 female, 6 male); TgAD(5 female, 6 male)] were HCHF-fed. *P < 0.05, **P < 0.01.

At the end of the 3-month period of consuming either CHOW alone (standard diet) or CHOW and HCHF (high carbohydrate high fat) diet, the average caloric intake per week of 6-month-old rats was higher in HCHF-fed rats (522 ± 99 kcal/week) than in CHOW-fed rats (284 ± 85 kcal/week). Similarly, in 12-month-old groups of rats, HCHF-fed rats consumed 518 ± 96 kcal/week whereas CHOW-fed rats ate 301 ± 72 kcal/week. When given *ad lib* access to HCHF foods, cohorts fed with HCHF showed significant increase in total calories consumed per week when compared to their respective CHOW only cohorts (6 months: 206 ± 17 kcal/week, *P < 0*.001; 12 months: 213 ± 33 kcal/week, *P < 0*.001), as shown in ***Figure 2A*** and ***Figure S1***. Compared to rats on CHOW only, HCHF-fed rats consumed more fats (6 months: 127 ± 29 kcal/week, *P = 0*.033; 12 months: 111 ± 27 kcal/week, *P = 0*.043) (***Figure 2B***), and more carbohydrates (6 months: 165 ± 32 kcal/week, *P = 0*.009; 12 months: 169 ± 29 kcal/week, *P = 0*.004) (***Figure 2C***).

***Figure 2E*** shows the weight of each group at baseline and after 3 months of either CHOW or HCHF diet.*** Figure 2F*** shows the percent weight gain relative to the reference weight (specified by the weight of the CHOW-fed male/female nTg rats) for each age group and sex (reference: CHOW-fed male/female nTg rats). The weight gain was higher in HCHF-fed groups (6 months: 27 ± 7% higher weight gain, *P < 0*.001; 12 months: 23 ± 5% higher weight gain, *P < 0*.001). Moreover, HCHF-fed female TgAD rats showed a weight increase that put them in the obese class 1 category, while all other HCHF-fed cohorts would be notionally classified as overweight (***Figure 2G***). At 12 months of age, HCHF-fed female rats had a higher increase in weight (by 15 ± 4% in nTg, *P = 0*.016; 29 ± 4% in TgAD, *P < 0*.001) compared to HCHF-fed male rats (***Figure 2G***). Sex segregated weight data are shown in ***Figure 2G***, with gray shading representing obese class 1 and class 2 based on categories defined for weight gain in humans 73.

### Blood glucose, BHB, and change in weight after overnight fasting

Blood glucose (***Figure S2A***) and BHB (***Figure S2B***) were measured at baseline, after fasting, and after 2DG injection. After 2DG injection, HCHF-fed cohorts exhibited higher blood glucose levels (above 28 mmol/L) compared to CHOW-fed cohorts (*P < 0*.001). HCHF-fed rats had higher blood BHB after the MRI scan (6 months: 1.5 ± 0.3 mmol/L, *P < 0*.001; 12 months: 0.8 ± 0.2 mmol/L, *P < 0*.001) compared to CHOW-fed littermates. After performing glucose and insulin tolerance tests, the rats demonstrated competent blood glucose regulation with an indication of potential glucose impairment developing in the HCHF diet group and TgAD rats (***Figure S3***).

### Attenuation of glucose uptake in HCHF-fed rats at 6 months vs its potentiation in HCHF-fed TgAD rats at 12 months

**Figure 3:**
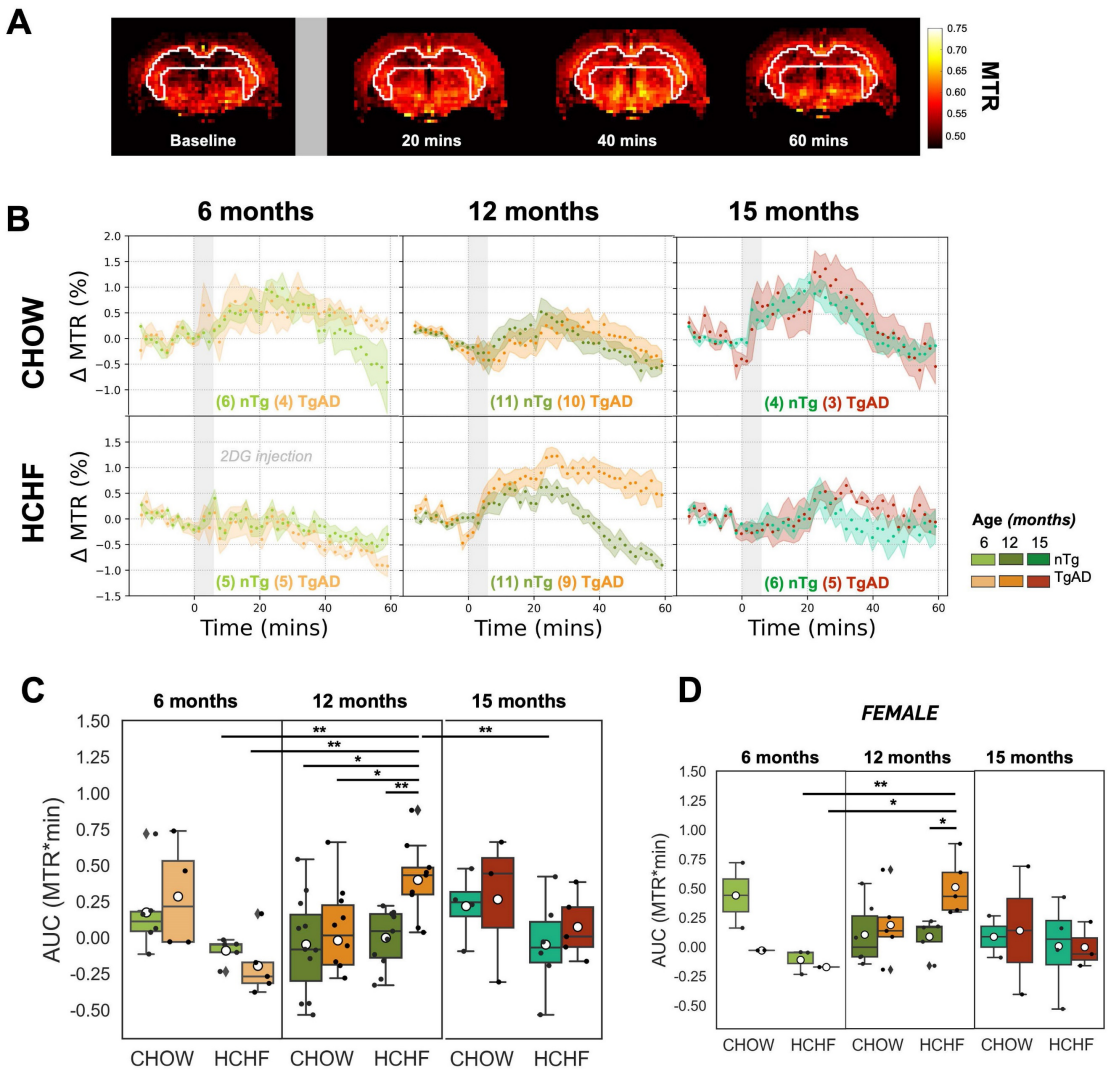
** HCHF attenuates hippocampal glucose uptake in presymptomatic AD, but elevates it in symptomatic AD**. **(A)** Representative time-resolved MTR change map at baseline and subsequent changes observed after the infusion of 2DG (indicated by the gray bar) at 20-minute intervals, illustrating the dynamics of 2DG uptake. **(B)** Dynamic CEST ΔMTR (mean and SEM) time courses in 6-, 12-, and 15-month-old nTg and TgAD cohorts fed with CHOW or HCHF diets. The time origin is the start of the 6-min 2DG infusion, indicated by the gray-shaded rectangle. **(C)** Area under the curve (AUC) of all scanned rats and **(D)** AUC of female rats over 60 minutes after the onset of 2DG infusion for 6-, 12-, and 15-month-old cohorts. There was a significant interaction of diet and genotype in 12-month-old cohorts (*P < 0*.001). At 6 months of age, there was a significant diet effect (*P < 0*.001) where HCHF-fed nTg rats (-0.09 ± 0.09 AUC units) had lower AUC units (by -153%) compared to CHOW-fed nTg (0.2 ± 0.3 AUC units); while HCHF-fed TgAD rats (-0.2 ± 0.2 AUC units) had lower AUC units (by -169%) compared to CHOW-fed TgAD rats (0.3 ± 0.4 AUC units). However, at 12 months of age, these contrasts were reversed: HCHF-fed nTg rats (-0.002 ± 0.20 AUC units) had higher AUC compared to CHOW-fed nTg (-0.1 ± 0.3 AUC units, by 2x), while HCHF-fed TgAD rats had higher AUC (0.4 ± 0.3 AUC units, by 20x) when compared to CHOW-fed TgAD (-0.02 ± 0.40 AUC units). Considering the HCHF-fed cohorts, 12-month-old TgAD rats (0.4 ± 0.3 AUC units) showed highest levels of hippocampal glucose uptake. They exhibited higher AUC compared to 12-month-old nTg rats (by 0.4 ± 0.1 AUC units, *P < 0*.001), to 6-month-old TgAD rats (by 0.6 ± 0.1 AUC units, by 4x, *P < 0*.001), and to 15-month-old nTg rats (by 0.5 ± 0.1, by 2x, *P < 0*.05). **(D)** After stratification by sex and pairwise comparisons, the differences across the groups remain evident when female rats were considered in isolation (initial uptake phase: age-gn-sex, *P < 0*.05; gn-sex, *P < 0*.001 | peak uptake phase: age-gn-sex, *P < 0*.05 and gn-sex, *P < 0*.05). In addition, the attenuation of glucose uptake of HCHF-fed 15-month-old TgAD rats compared to 12-month-old TgAD rats was driven by females (initial uptake phase: sex, *P < 0*.05), but not statistically significant after pairwise comparison. At 6 months of age, n=20, where n=9 [nTg(1 female, 2 male); TgAD(4 female, 2 male)] are CHOW-fed and n=11 [nTg (3 female, 2 male); TgAD (2 female, 4 male)] are HCHF-fed. At 12 months of age, n=41, where n=21 [nTg (6 female, 5 male); TgAD (5 female, 5 male)] were CHOW-fed and n=20 [nTg (5 female, 6 male); TgAD (5 female, 4 male)] were HCHF-fed. At 15 months of age, n=18, where n=7 [nTg (2 female, 2 male); TgAD (2 female, 1 male)] were CHOW-fed and n=11 [nTg (4 female, 2 male); TgAD (3 female, 2 male)] were HCHF-fed. *P < 0.05, **P < 0.01.

The estimation of hippocampal glucose uptake was done by quantifying the area under the curve of the dynamic CEST signal over an hour following 2DG administration, with representative MTR maps shown in ***Figure 3A*** and hippocampal CEST signal time courses shown in ***Figure 3B****.* In light of the kinetics of glucose uptake 44, we followed our earlier work 44 and divided the 60-min glucose uptake time courses into three 20-min segments (as shown in ***Figure S4***), corresponding to the initial uptake, the peak uptake, and the washout phases.

In presymptomatic AD (at 6 months of age), as shown in ***Figure 3C***, HCHF-fed rats, both nTg and TgAD, showed lower hippocampal glucose uptake compared to littermates fed CHOW only (60-min | diet effect, *P < 0*.001; initial uptake phase | diet effect, *P < 0*.01; peak uptake phase | diet effect, *P < 0*.001). This attenuation was larger in TgAD (CHOW-TgAD: 0.3 ± 0.4 AUC units; HCHF-TgAD: -0.2 ± 0.2 AUC units, by -153%) than in nTg (CHOW-nTg: 0.2 ± 0.3 AUC units; HCHF-nTg: 0.1 ± 0.1 AUC units, by -79%) (60-min | diet-genotype, *P < 0*.001). Notwithstanding, low subject numbers by sex in the 6-month-old cohort, the attenuation of glucose uptake of HCHF-fed cohorts (when compared to CHOW-fed cohorts) were sex dependent (60-min | diet-genotype-sex, *P < 0*.01; initial uptake phase | diet-genotype-sex, *P < 0*.001, diet-sex,* P < 0*.05; peak uptake phase | diet-genotype-sex, *P < 0*.01, diet-sex, *P < 0*.05) (***Figure S5***).

However, in symptomatic AD (at 12 months of age), as shown in ***Figure 3C***, HCHF diet led to an enhancement of glucose uptake in TgAD - but not in nTg - rats (60-min | diet-genotype, *P < 0*.001; genotype, *P < 0*.001). HCHF-consuming TgAD rats (0.4 ± 0.2 AUC units) had the highest glucose uptake among all cohorts; their glucose uptake was 20x higher than that of CHOW-fed TgAD rats (-0.02 ± 0.4 AUC units). Notably, after stratification by sex, the differences observed after pairwise comparisons across the different groups remain evident in the females (***Figure 3D, Figure S5***). At 15 months of age, the hippocampal glucose uptake was not significantly different among the groups. In summary, the enhancement of glucose uptake of HCHF-fed 12-month-old TgAD rats was observed only in the hippocampus and diencephalon (and was more prominent in the hippocampus), ***Figure S6***. The comparison of glucose uptake in other regions, across all age groups, can be found in ***Figure S6***.

### Detrimental cerebrovascular effects of HCHF in 6-month-old animals and its cerebrovascular benefits in 12-month-old TgAD rats

**Figure 4:**
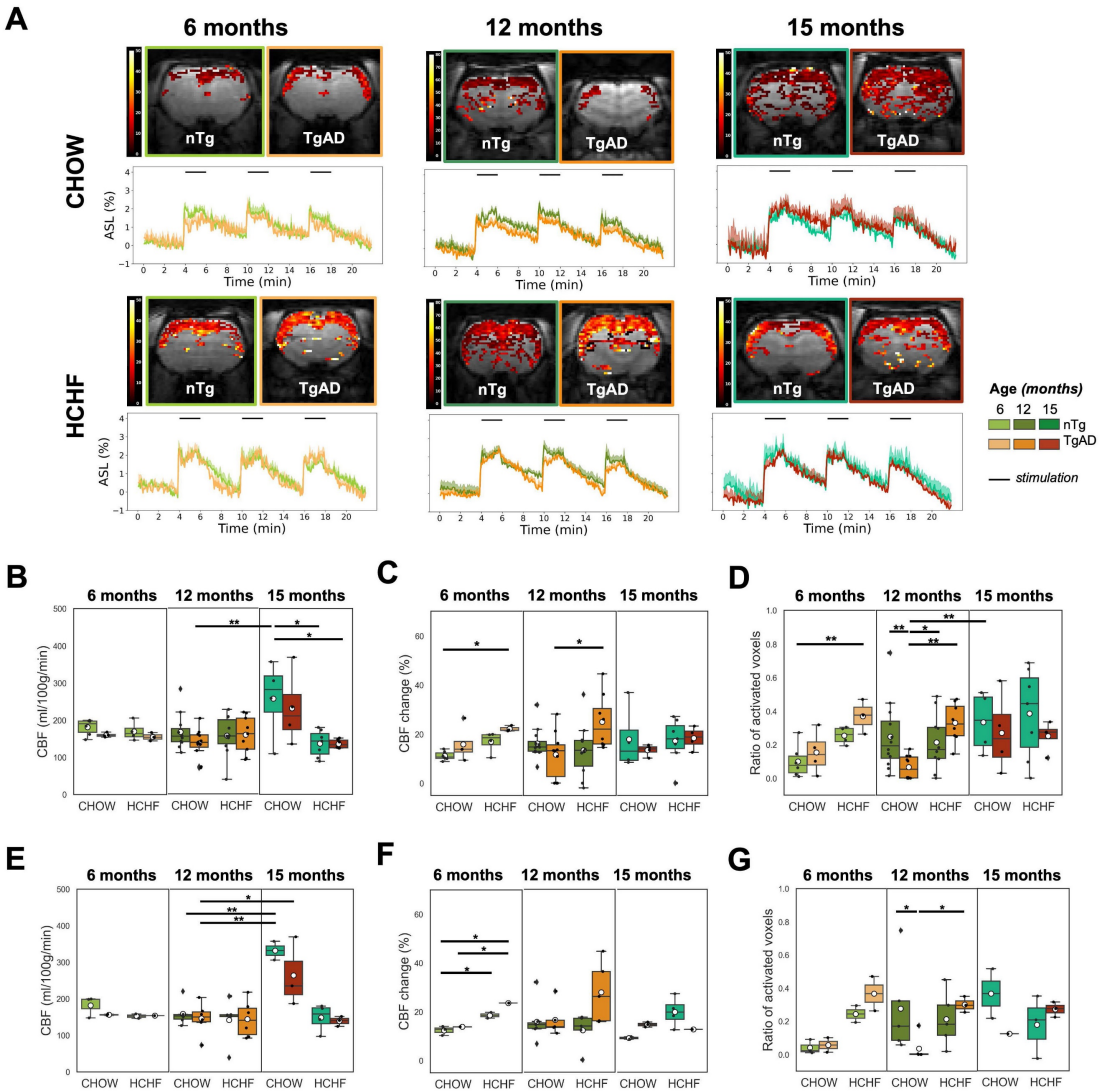
** HCHF elevates functional hyperemia in presymptomatic and symptomatic AD; and decreases resting perfusion in presymptomatic AD. (A)** Representative maps of CBF changes, and average hippocampal ASL time courses normalized by the mean resting ASL in response to bilateral forepaw stimulation. **(B)** The resting CBF of cohorts at 6 and 12 months were indistinguishable from each other. However at 15 months, lower resting CBF in HCHF-fed rats (nTg: 136 ± 36 ml/100g/min; TgAD: 136 ± 12 ml/100g/min) by 47% in nTg and 41% in TgAD rats was observed compared to CHOW-fed rats (nTg: 258 ± 107 ml/100g/min; TgAD: 232 ± 100 ml/100g/min) mainly in females (diet, *P < 0.001*; diet-sex, *P < 0.05*). At 6 months, HCHF-fed rats had significantly lower resting CBF compared to CHOW-fed rats (diet, *P < 0*.001). **(C)** At 6 months, HCHF-fed rats (nTg: 17 ± 4%; TgAD: 22 ± 1%) showed a greater increase in CBF in response to forepaw electric stimulation by 51% in nTg and 40% in TgAD compared to CHOW-fed rats (nTg: 11 ± 2%; TgAD: 16 ± 7%) (diet, *P < 0.01*). However, the effect of HCHF in 12 and 15 months of age was genotype specific (12 months: diet-genotype, *P < 0.001;* 15 months: diet-genotype-sex, *P < 0.05*). The CBF response to forepaw stimulation of HCHF-fed nTg rats (12 months: 13 ± 11%; 15 months: 17 ± 9%) are lower (by 17% in 12 months and 4% in 15 months) compared to CHOW-fed nTg rats (12 months: 16 ± 6%; 15 months: 18 ± 13%) while HCHF-fed TgAD rats (12 months: 25 ± 11%; 15 months: 18 ± 5%) have higher CBF response (by 114% in 12 months and 37% in 15 months) compared to CHOW-fed TgAD rats (12 months: 12 ± 9%; 15 months: 13 ± 2%). **(D)** At 6 months of age, HCHF-fed rats had a larger area of activation by 154% in nTg and by 141% in TgAD rats when compared to CHOW-fed rats (diet, *P < 0*.001); whereas HCHF-fed TgAD rats (0.37 ± 0.10) had significantly higher area of activation compared to CHOW-fed nTg rats (0.1 ± 0.1, *P < 0*.001). At 12 months of age, there was a significant interaction of diet and genotype (*P < 0*.001): HCHF-fed nTg (0.2 ± 0.2) rats exhibited a trend toward lower area of activation by 14% when compared to CHOW-fed nTg (0.3 ± 0.2). In contrast, HCHF-fed TgAD rats (0.3 ± 0.1) showed a trend of higher area of activation by 400% compared to that of CHOW-fed TgAD rats (0.07 ± 0.07). At 15 months of age, there was no difference in the areas of activation between cohorts. **(E)** The observed difference in resting perfusion and **(F)** CBF change was driven by female rats while the **(G)** area of activation was more prominent in male rats. In evaluating resting perfusion at 6 months of age, n=17, where n=10 [nTg (3 female, 3 male); TgAD (2 female, 2 male)] were CHOW-fed and n=7 [nTg (2 female, 2 male); TgAD (1 female, 2 male)] were HCHF-fed. At 12 months of age, n=41, where 22 [nTg (6 female, 5 male); TgAD (6 female, 5 male)] were CHOW-fed and 19 [nTg (5 female, 5 male); TgAD (6 female, 3 male)] were HCHF-fed. At 15 months of age, n=21, where n=8 [nTg (2 female, 2 male); TgAD (3 female, 1 male)] were CHOW-fed and n=13 [nTg (4 female, 3 male); TgAD (3 female, 3 male)] were HCHF-fed. *P < 0.05, **P < 0.01.

The voxelwise magnitude of pCASL signal was averaged to estimate cerebrovascular reactivity, i.e., the increase in cerebral blood flow, in response to forepaw stimulation over the imaging slice. Representative activation maps and CBF time course change in response to bilateral forepaw stimulation of CHOW- and HCHF-fed nTg, and TgAD cohorts at 6, 12, and 15 months, were shown in ***Figure 4A*** At 6 months of age, there was a diet effect (*P < 0*.001) on resting CBF: HCHF-fed rats (nTg: 170 ± 26 ml/100g/min, TgAD: 155 ± 11 ml/100g/min) had lower (nTg: by 6%, TgAD: by 3%) resting CBF compared to CHOW-fed rats (nTg: 181 ± 22 ml/100g/min, TgAD: 160 ± 6 ml/100g/min). At 12 months of age, resting CBF among cohorts, fed with either diet, was indistinguishable (167 ± 48 and 136 ± 39 ml/100g/min for CHOW-fed nTg and TgAD rats; 157 ± 55 and 159 ± 50 ml/100g/min for CAF-fed nTg and TgAD rats) (***Figure 4B)***. After pairwise comparison, HCHF-fed female rats showed a trend toward lower resting CBF compared to male rats in both nTg and TgAD cohorts. With prolonged exposure to the HCHF diet (at 15 months), HCHF-fed cohorts had lower resting CBF compared to CHOW-fed cohorts (diet effect, *P < 0.001*), a contrast driven by female rats (diet-sex, *P < 0.05*) (***Figure 4E, Figure S7A)***. For either diet, male rats (CHOW-nTg: 184 ± 106 ml/100g/min; CHOW-TgAD: 135 ml/100g/min; HCHF-nTg: 120 ± 32 ml/100g/min; HCHF-TgAD: 133 ± 13 ml/100g/min) had lower resting perfusion when compared to female rats (CHOW-nTg: 332 ± 36 ml/100g/min; CHOW-TgAD: 264 ± 95 ml/100g/min; HCHF-nTg: 148 ± 38 ml/100g/min; HCHF-TgAD: 139 ± 14 ml/100g/min) (***Figure S7A,D***). The absolute resting perfusion was not distinguishable between groups in any of the other brain regions (cortex, S1FL) (***Figure S8***).

At 6 months, the CBF response to forepaw stimulation of HCHF-fed rats (nTg: 17 ± 4%, TgAD: 22 ± 1%) were higher (by 51% in nTg and 40% in TgAD; diet, *P < 0*.01) compared to CHOW-fed rats (nTg: 11 ± 2%, TgAD: 16 ± 7%). At 12 and 15 months, the CBF response to forepaw stimulation (12 months: diet-genotype, *P < 0.001;* 15 months: diet-genotype-sex, *P < 0.05*) of HCHF-fed nTg rats (12 months: 13 ± 11%; 15 months: 17 ± 9%) was lower (by 17% in 12 months and 4% in 15 months) compared to that of CHOW-fed nTg rats (12 months: 16 ± 6%; 15 months: 18 ± 13%); while HCHF-fed TgAD rats (12 months: 25 ± 11%; 15 months: 18 ± 5%) had higher CBF response (by 114% in 12 months and 37% in 15 months) compared to that of CHOW-fed TgAD rats (12 months: 12 ± 9%; 15 months: 13 ± 2%) (***Figure 4C, Figure S7B***).

At 6 months, the activation area of HCHF-fed rats (nTg: 0.25 ± 0.05, TgAD: 0.37 ± 0.10) was greater by 154% in nTg and by 141% in TgAD rats when compared to CHOW-fed rats (nTg: 0.1 ± 0.1; TgAD: 0.15 ± 0.13) (diet, *P < 0*.01) (***Figure 4D***). The 6-month-old HCHF-fed TgAD rats also had larger area of activation (0.37 ± 0.10) by 45% compared to HCHF-fed nTg (0.25 ± 0.05) and higher by 269% compared to CHOW-fed nTg (0.1 ± 0.1) (genotype, *P < 0*.001) (***Figure 4D***). However at 12 months, the effect of HCHF was genotype specific (diet-genotype, *P < 0*.001): TgAD rats (0.33 ± 0.12) showed a trend toward higher (approximately 400%) area of activation while nTg rats showed a trend toward lower (approximately -14%) area of activation with HCHF diet when compared to the CHOW only consuming counterparts (nTg: 0.25 ± 0.21; TgAD: 0.07 ± 0.07) (***Figure 4D***). In general, the interaction of diet and genotype was observed in functional hyperemia within several brain regions (cortex, S1FL, globally) (***Figure S8***). At 15 months of age, there was no difference in the areas of activation between CHOW-fed (nTg: 0.33 ± 0.19; TgAD: 0.27 ± 0.24) and HCHF-fed cohorts (nTg: 0.39 ± 0.27; TgAD: 0.25 ± 0.09) (***Figure 4D***). Comparing 12- and 15-month-old cohorts, CHOW-fed TgAD rats had lower areas of activation compared to that of CHOW-fed nTg rats (genotype, *P < 0.001*). Conversely, the effect of HCHF diet on area of activation differed at 15 months compared to 12 months (age-genotype, *P = 0.059*) of age: HCHF-fed TgAD rats showed a trend toward lower area of activation compared to that of HCHF-fed nTg rats at 15 months, whereas the opposite contrast was seen at 12 months.

### Elevated hippocampal lectin density of TgAD rats and its attenuation with prolonged HCHF diet

**Figure 5:**
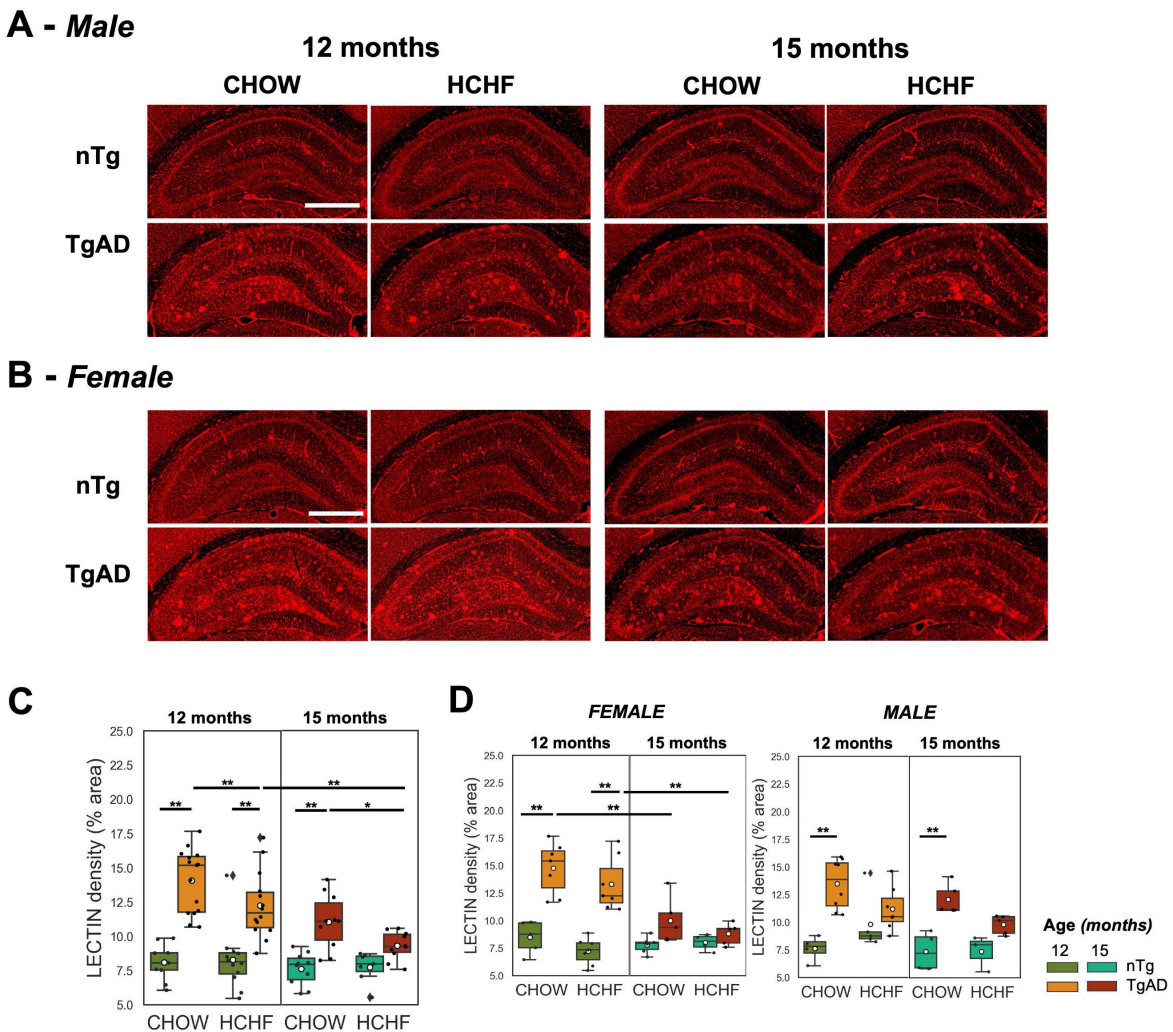
** Prolonged HCHF diet attenuates lectin density in TgAD rats. (A-B)** Representative tomato lectin-Texas Red-stained images of hippocampal capillaries in CHOW- and HCHF-fed nTg and TgAD 12-month-old rats. **(C)** 12-month-old TgAD rats had higher lectin density compared to nTg rats. HCHF attenuated the lectin density in TgAD rats, with further decreases seen with prolonged HCHF diet. **(D)** Both male and female TgAD and nTg rats had comparable lectin density at 15 months. At 12 months, n=50, where n=26 [nTg (7 female, 5 male), TgAD (7 female, 7 male)] were fed with HCHF diet and n=24 [nTg (5 female, 4 male), TgAD (7 female, 8 male)] were fed with CHOW. At 15 months, n=37, where n=17 [nTg (4 female, 3 male), TgAD (5 female, 5 male)] were fed with HCHF and n=20 [nTg (6 female, 4 male), TgAD (5 female , 5 male)] were fed with CHOW. Scale bar: 1mm. *P < 0.05, **P < 0.01.

Hippocampal vascular density of CHOW- and HCHF-fed nTg and TgAD 12-month-old rats was measured via tomato lectin-Texas Red staining (representative images in (***Figure 5A-B)*** and quantified (***Figure 5C***)***.*** Hippocampal vascular density of 12-month-old TgAD rats was greater by 74% (14 ± 2%) compared to that of nTg rats (8 ± 1%) in CHOW-fed cohorts (*P < 0*.001) and in HCHF diet-fed cohorts (nTg: 8 ± 2%, TgAD: 12 ± 2%, *P < 0*.001) (***Figure 5C***). Segregated by sex, the difference in lectin density between HCHF-fed nTg and TgAD rats was only observed in female rats (genotype-sex, *P < 0*.01) (***Figure 5D***). With prolonged HCHF diet, vascular density of 15-month-old HCHF diet-fed TgAD rats significantly decreased by 24% (12 ± 2% at 12 months, 9 ± 1% at 15 months, *P < 0*.001) to the level of HCHF-fed nTg rats (8 ± 1%). At 15 months, HCHF-fed TgAD rats (8 ± 1%) showed a 16% lower hippocampal vascular density, when compared to CHOW-fed TgAD (11 ± 2%) (diet-genotype, *P < 0.05*).

### Normalization of elevated hippocampal AQP4 density in HCHF-fed male TgAD rats at 12 months, and increased hippocampal CAA density in HCHF-fed female TgAD rats at 15 months

**Figure 6:**
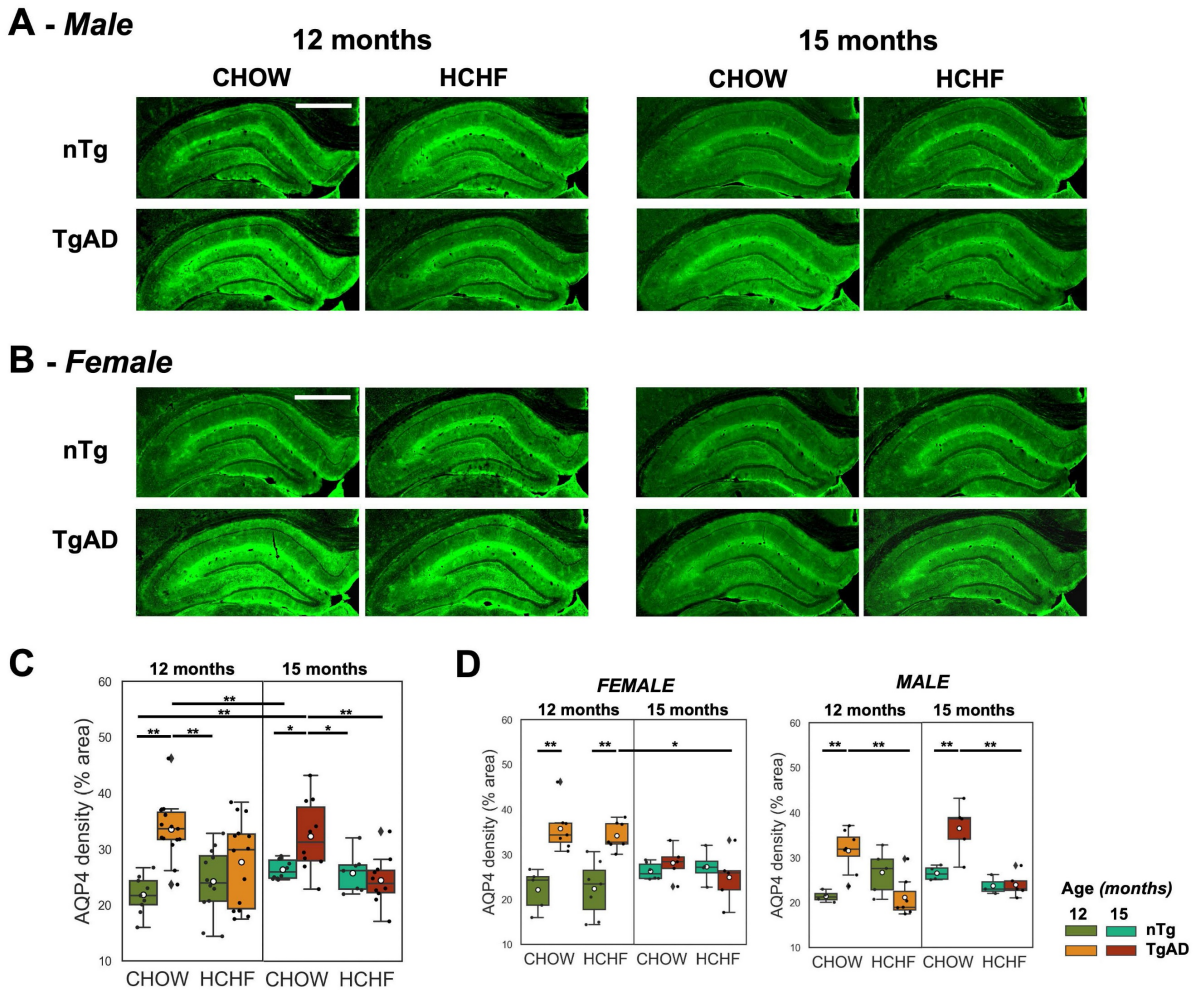
** Prolonged HCHF diet rescued elevated hippocampal AQP4 density in symptomatic AD. (A-B)** Representative stained images of AQP4 in both CHOW- and HCHF-fed 12- and 15-month-old **(A)** male and **(B)** female rats. **(C)** At 12 months of age, there was a significant interaction of genotype and diet (*P < 0*.001), whereby AQP4 density was elevated in TgAD rats, but rescued with HCHF diet and maintained with extended HCHF diet at 15 months. **(D)** The attenuation of hippocampal AQP4 density of HCHF-fed TgAD rats (28 ± 8%) compared to CHOW-fed TgAD rats (34 ± 5%, *P < 0*.001) at 12 months was driven by males (diet-genotype-sex, *P < 0.01*; genotype-sex, *P < 0.001*). With prolonged exposure to the HCHF diet, there was a female-driven attenuation of AQP4 density (age-genotype-sex, *P < 0*.001; genotype-sex, *P < 0*.001). At 15 months, CHOW-fed male TgAD rats maintained an elevated hippocampal AQP4 density (age-genotype-sex, *P < 0.05*). At 12 months, n=50, where n=26 [nTg (7 female, 5 male), TgAD (7 female, 7 male)] were fed with HCHF diet and n=24 [nTg (5 female, 4 male), TgAD (7 female, 8 male)] were fed with CHOW. At 15 months, n=37, where n=17 [nTg (4 female, 3 male), TgAD (5 female, 5 male)] were fed with HCHF and n=20 [nTg (6 female, 4 male), TgAD (5 female, 5 male)] were fed with CHOW. Scale bar: 1mm. *P < 0.05, **P < 0.01.

**Figure 7:**
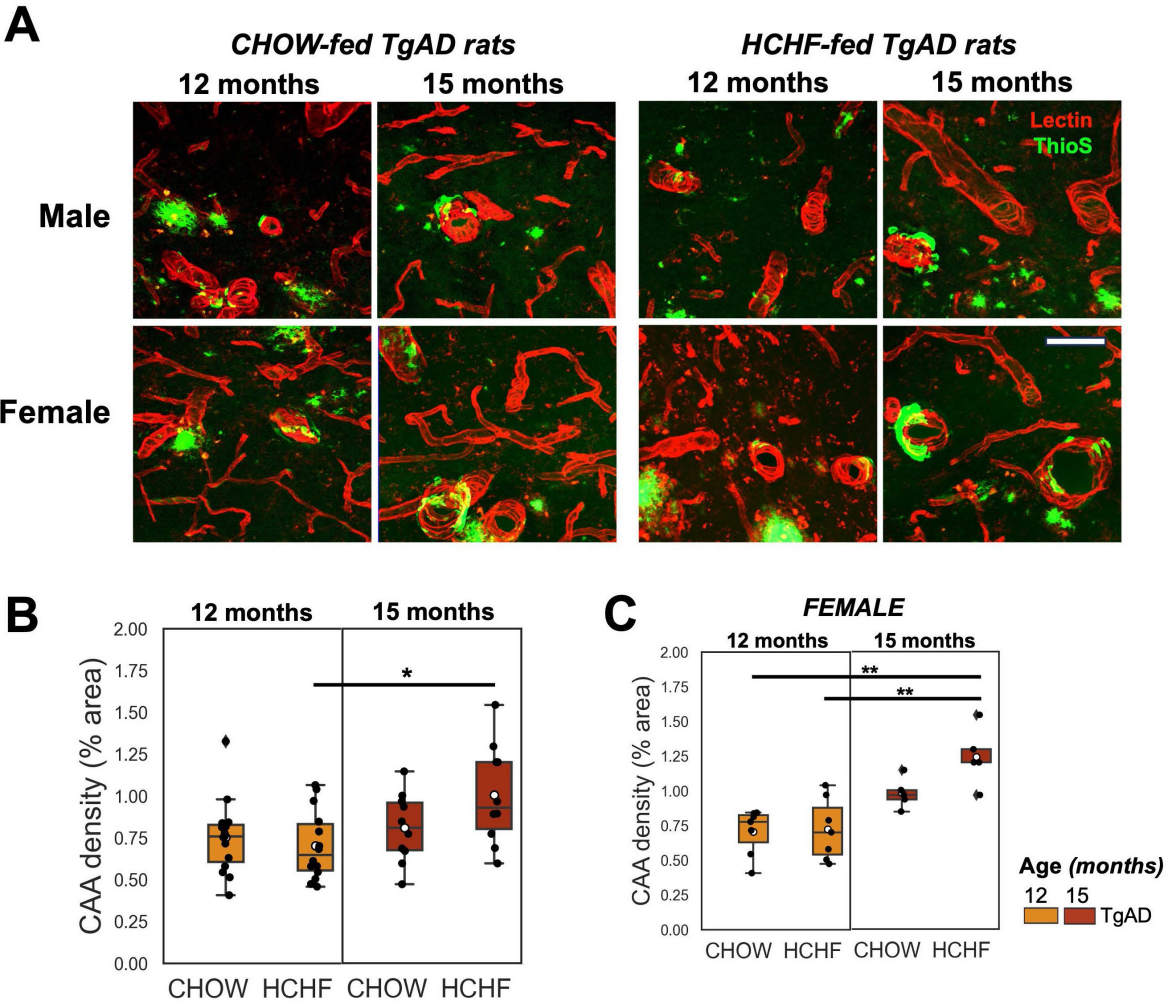
** Prolonged HCHF exacerbated cerebral amyloid angiopathy in females. (A)** Representative stained (tomato lectin-Texas Red) images of hippocampal CAA in TgAD rats fed with CHOW and HCHF diet for 3 months (12-month-old rats) and 6 months (15-month-old rats). **(B)** Quantification of hippocampal CAA density showed a significant interaction of age and sex (*P < 0*.01) and a significant effect of age (*P < 0*.001). **(C)** the increase in CAA coverage with age was driven by female rats (sex, *P < 0.001*). At 12 months, n=29, where n=14 (7 female, 7 male) was fed with HCHF diet and n=15 (7 female, 8 male) was fed with CHOW. At 15 months, n=20, where n=10 (5 female, 5 male) was fed with HCHF and n=10 (5 female, 5 male) was fed with CHOW. Scale bar: 0.07 mm. *P < 0.05, **P < 0.01.

Hippocampal AQP4 was evaluated as a putative metric of BBB integrity. The representative stained images of AQP4 and the corresponding quantification is shown in ***Figure 6A*** and ***Figure 6B***. In the symptomatic stage of AD (12 months), there was a significant interaction of diet and genotype on AQP4 density (*P < 0*.001) (***Figure 6C***). CHOW-fed TgAD rats had 54% increase in AQP4 density compared to CHOW-fed nTg rats (TgAD: 34 ± 5%, nTg: 22 ± 3%,* P < 0*.001). This elevation, frequently interpreted as a manifestation of BBB impairment, was not present in the HCHF consuming TgAD rats (***Figure 6C***). Stratifying by sex, only male TgAD rats showed attenuation of AQP4 density when fed with HCHF (11 ± 2%, *P < 0*.01) while female rats maintained an elevated AQP4 density (***Figure 6D***). With prolonged exposure to HCHF diet, female TgAD rats did show attenuation of hippocampal AQP4 density (significant interactions of diet-genotype-sex: 20 ± 5%, *P < 0*.001; genotype-sex: -17 ± 3%, *P < 0*.001; age-genotype: -14 ± 4%, *P < 0*.001; and genotype: 12 ± 2%, *P < 0*.001) and an accompanying increase in cerebral amyloid angiopathy (significant age and sex interaction: -0.4 ± 0.2%, *P = 0*.004; and age effect: 0.5 ± 0.1%, *P < 0*.001) (***Figure 7C***). At 15 months, CHOW-fed TgAD rats (32 ± 6%) maintained an elevated hippocampal AQP4 density compared to CHOW-fed nTg (26 ± 2%) (diet-genotype, *P < 0.001*: age-genotype, *P < 0.05*), evidently in male rats (age-genotype-sex, *P < 0.05*).

## Discussion

Dietary regimens have been extensively studied as a method of abating the progression of Alzheimer's Disease and dementia 38,74-78. Although some diets have shown promise 79-84, these investigations have been challenged by the complexity of the metabolic pathways (as reviewed by Bhat *et al.* 85), the heterogeneity in the pathological changes comprising AD 86, and the low adherence to interventions in humans 4. In the current study, we examined the effect of high carbohydrate high fat (HCHF) diet-induced obesity on hippocampal glucose uptake and hemodynamics in presymptomatic (6 months of age) vs. symptomatic (12 months of age) transgenic Fischer 344 AD rats, via non-invasive imaging techniques. Regardless of age, rats fed the HCHF diet showed higher caloric intake and greater weight gain than CHOW-fed nTg rats, with female rats exhibiting more pronounced weight gain than male rats. Regardless of age, obesity had harmful effects on neurophysiology in nTg rats. In contrast to its effects on normal aging, the effects of obesity were disease-stage specific. While obesity in the presymptomatic stage of the disease had pernicious effects, as manifested by attenuated hippocampal glucose uptake and decreased resting cerebral perfusion; obesity during the symptomatic stage of AD prevented the disease-dependent decrease in hippocampal glucose uptake and cerebrovascular reactivity, despite unabated progression in amyloid and tau pathologies, as seen by our accompanying study in the same animals 46. The 12-month-old TgAD rats showed elevated hippocampal glucose uptake and cerebrovascular reactivity in relation to that of CHOW-fed 12-month-old nTg; and their brain blood flow was indistinguishable from that of 12-month-old CHOW-fed nTgs. With prolonged exposure to HCHF (over 6 vs. 3 months) the hippocampal glucose uptake of the TgAD rats was indistinguishable from that of their nTg littermates. The statistical models were rerun to include body weight as a covariate and measured the likelihood ratio p-value to test whether the addition of weight variable improves the model fit. We have not found a significant effect of body weight in addition to the diet, given the binomial distribution of the body weights (whereby HCHF fed animals were all significantly higher in body weight than their CHOW fed counterparts).

### HCHF effects in presymptomatic AD

The reduction of hippocampal glucose uptake and resting perfusion in 6-month-old rats can be interpreted as detrimental, as the degree of hippocampal hypometabolism has been shown to be correlated with cognitive decline 87. In support of the detrimental effects of presymptomatic stage HCHF exposure, Anderson *et al.*, showed that Western diet-induced obesity in 6.5-month-old TgF344-AD rats leads to impairment in spatial working memory and increased soluble Aβ_42_ level in dorsal hippocampus 36. The lack of genotype effect is expected in light of our 6-month old TgF344-AD rats having minimal amyloid 41, and only starting to show subtle cognitive dysfunction 40, similarly to some other TgF344-AD colonies 88,89. When TgAD rats were fed with HCHF diet, the observed attenuation in glucose uptake was greater compared to nTg rats. This difference may be indicative of impaired neuronal function, possibly resulting from inefficient glucose uptake and utilization. Anderson *et al.*, have demonstrated that 6.5-month-old TgF344-AD rats exhibit tricarboxylic acid cycle (TCA) enzyme dysregulation, modest down-regulation of ETC (electron transport chain) complexes, and decreased expression of *Slc16a3*/MCT4 36. After administration of Western diet, mitochondrial dysregulation is heightened; ETC complexes downregulation is exacerbated; and *Slc16a3*/MCT4 expression is further decremented, all leading to compromised ATP (adenosine triphosphate) synthesis, accelerated ROS (reactive oxygen species) generation, and altered glycolytic flux 36. The findings of Anderson *et al.*, on the effects of Western diet on 6.5-month-old TgF344-AD rats support the greater attenuation of glucose uptake in our HCHF-fed 6-month-old TgAD rats. Notwithstanding, HCHF diet fed animals exhibited potentiated functional hyperemia to bilateral forepaw electric stimulation, a possible compensation for the decreased baseline CBF 44. The adverse impact of obesity induced by HCHF diet in 6-month-old rats on neurophysiological state may underlie findings in the human population, where obesity during midlife is associated with increased risk and progression of AD 31.

### HCHF effects in symptomatic AD

At 12 months of age, obesity led to enhancement of glucose uptake and functional hyperemia spread in (primarily female) TgAD rats, while still being detrimental to nTg animals. We posit that these changes are beneficial because higher metabolic activity enables greater levels of neuronal activity in the AD-challenged hippocampus. This notion is supported by our accompanying work 46 that demonstrates a decrease in microglia/macrophages (on Iba-1 (Ionized Calcium Binding Adaptor Molecule 1)) and stable astrocytic activity (on GFAP (glial fibrillary acidic protein)) in HCHF-fed 12-month old TgAD rats: the metabolic elevation observed here is thus not related to heightened neuroinflammation. Mitochondrial complexes are protein assemblies essential in cellular respiration and energy production. Denver *et al.* reported down regulation of mitochondrial complex I (NADH: ubiquinone oxidoreductase) in the hippocampus of 16-18-month-old TgF344-AD rats indicating bioenergetic deficit 47, which may be compensated by increased consumption of energy substrates with HCHF. Their findings support the notion that the AD brain is metabolically challenged; it may thus benefit from increased availability of energy substrates, presently achieved via the administration of HCHF food items. Our accompanying study lends further support to this assertion by showing that 3 months of HCHF consumption results in significant improvement of the 12-month-old female TgAD rats' executive function, despite neuronal loss 46. Further, this amelioration of spatial learning deficits was not attributable to reductions in amyloid plaques or tau pathology 46. Hippocampal volume measurements on MRI across all the groups were indistinguishable (***Figure S9***) indicating that the deficit in spatial learning cannot be attributed to atrophy. Fowler *et al.* also reported an absence of atrophy up to 15 months in TgF344-AD rats using MRI-based voxelwise morphometry, with atrophy becoming apparent from 16 to 18 months 90. Other MRI studies have reported no atrophy even up to 18 months of age 91. Minhas *et al.* also demonstrated that restoring hippocampal glucose metabolism in mice models of AD pathology (5XFAD and P301S) restores spatial memory 92. Conversely, ketogenic diet and calorie restriction failed to improve cognition or motor behavior in 12-month-old TgF344-AD rats 75. We thus speculate that the positive effects observed in our study were specifically linked to overconsumption.

Vascular density was measured to evaluate if HCHF caused morphological changes in the cerebrovasculature. We observed a sex contrast irrespective of diet: the capillary density of female TgAD rats was elevated compared to that of female nTg rats, but there was no such contrast in males. Despite these morphological changes in brain capillaries, the resting perfusion was comparable across all groups, suggesting that the variation in glucose uptake was not due to the alteration of glucose delivery at rest. The sustained resting blood flow in HCHF TgAD rats was thus likely driven by functional rather than anatomical alterations, e.g., the vascular drive from the metabolically more active tissue of HCHF fed rats.

Cerebrovascular reactivity to elevation in neuronal activity is a standard measure of cerebrovascular functioning 93. Cerebrovascular reactivity, as indicated by the area of activation, was compromised in TgAD rats compared to nTg rats 44. However, this impairment was absent in HCHF-fed TgAD rats, particularly in females. Impairment in functional hyperemia is observed in male C57BL6/J mice after feeding with a high caloric Western diet for 3 weeks 94. In addition to the cerebrovascular reactivity, the blood-brain barrier (BBB) may play a role in the delivery and uptake of energy substrates. CHOW-fed TgAD rats had a higher hippocampal density of integral structural protein of BBB (AQP4), previously shown to correlate to higher BBB permeability 95. With the HCHF diet, this AQP4 level elevation was abolished in male rats, their ensuing AQP4 levels being similar to those of CHOW- and HCHF-fed nTg rats. While AQP4 levels in female TgAD rats remained elevated with HCHF diet, HCHF-fed female TgAD rats exhibited increased myelin level 46, a change likely to have beneficial effects on neurotransmission. Increase expression of AQP4 around beta amyloid plaques in animal models of AD 96,97. Compared to 3xTgAD mice fed with standard chow diet, those on a high fat/cholesterol diet exhibit a trend of increased AQP4 expression. Of note, post-mortem analysis of AD patients show heightened expression of AQP4, suggesting disrupted BBB permeability that may result in reduced amyloid beta clearance and entry of neurotoxic and pro-inflammatory molecules 98-100, which can ultimately lead to changes in metabolic activity and neuronal function.

Overall, among 12-month-old HCHF-fed TgAD rats, females exhibited a higher increase in weight, heightened hippocampal glucose uptake and functional hyperemia, along with higher capillary and myelin density, but also higher AQP4 density compared to male rats. Aside from female rats gaining more weight after feeding with HCHF diet compared to male rats, HCHF diet might affect estrous cycle 101, glucose clearance in females 102, and cyclical increases in follicle-stimulating hormone 103, all contributing to the observed sex differences.

An additional cohort was exposed to prolonged HCHF diet for 6 months to assess whether the observed enhancement in glucose uptake in symptomatic AD rats is a transient compensatory mechanism, similar to the hypermetabolism observed in a mouse model of early AD stage (7-month-old Tg2576), without significant alterations in hemodynamics 104. With prolonged (6-month long) HCHF exposure, fifteen-month-old TgAD rats showed attenuated hippocampal glucose uptake compared to 12-month-old TgAD rats (after 3 months of HCHF) demonstrating that the enhanced glucose uptake was transient at early AD stage at 12 months. The lack of contrast in hippocampal glucose uptake between TgAD and nTg rats at 15 months of age may be due to increased neuronal loss after 6 months of HCHF 46. Notwithstanding, at 15 months of age, resting CBF, functional hyperemia, and hippocampal glucose uptake of HCHF-fed TgAD were indistinguishable from those of HCHF-fed nTg rats. However, resting CBF of CHOW-fed 15-month-old cohorts was higher than that of HCHF-fed rats and was primarily observed in female rats. Wang *et al.* showed that female Sprague-Dawley rats have higher basal myogenic tone, lower distensibility, reduced VSMC contractility, thinner vascular wall, smaller inner diameter, and increased elastin and collagen content 105. High levels of estrogens with increased prostacyclin production in women is speculated to result in higher CBF due to higher capacity for vasodilatory response to CO_2 106-108_. Sex-difference in hemoglobin level in our animal model of AD could also contribute to the observed difference in CBF as hemoglobin level. Higher CBF velocity is observed in younger women (age < 55 years) compared to young men, then reversed at age > 85 where the trend of sex-difference in CBF with age is inversely correlated with hemoglobin level 109. These attributes likely contribute to sex differences in resting CBF at 12 and 15 months of age, but future work is needed to elucidate the mechanisms underlying sex contrast in brain blood flow over the lifespan. The increase in perfusion is also observed in normal aging, MCI, and early AD stages that may be compensatory 110-112. In addition, hippocampal capillary and AQP4 densities were indistinguishable between HCHF-fed TgAD and nTg rats despite the AD pathology progression at 15 months (specifically, the increase in cerebral amyloid angiopathy in females). The high AQP4 density of CHOW-fed TgAD rats compared to those of CHOW-fed nTg rats, HCHF-fed TgAD, and HCHF-fed nTg suggests that there might be alterations in the blood brain barrier's integrity and function brought about by the transgenes, that are transiently normalized by diet.

Hypometabolism and compensatory hypermetabolism are also observed in human studies. In healthy adults, cerebral glucose metabolism is negatively correlated with BMI and positively correlated with cognitive function 113. Hypometabolism is observed in healthy adults with high BMI 113, MCI, and AD subjects 114-117. In MCI and AD subjects, attenuation of cerebral metabolic rate for glucose by 20-30% is observed compared to healthy controls using PET 114-117. On the other hand, hypermetabolism is observed in morbidly obese women 118 and MCI patients 119,120. As the cognitive function of morbidly obese women with hypermetabolic brain is preserved, this hypermetabolism has been suggested to be a compensatory mechanism 118. The hypermetabolism in MCI subjects is also described to be a transient compensatory mechanism and is followed by a hypometabolism as Aβ load worsens 119. Obese ApoE(+) subjects (> 70 years of age) have higher cerebral glucose metabolism, superior cognitive performance at baseline, and less cognitive decline over time in comparison to ApoE(+) overweight and normal weight subjects 120. A positive correlation is also demonstrated between nutritional status (BMI and waist to height ratio) and regional cerebral glucose metabolism in early AD 121. In addition, meta-analysis shows that BMI is negatively associated with CBF 122-124. Arterial spin-labeling MR imaging study also shows regional hypoperfusion in the brain of AD patients 125,126 and attenuated functional hyperemia to visual stimulation in early AD patients when compared to healthy control 127. However, an increase in brain fractional perfusion, measured using noninvasive intravoxel incoherent motion MRI technique, is observed in obese participants and is associated with better working memory performance 122.

## Conclusion

Most of the studies on diet intervention in experimental models of AD to date have described the onset of diet intervention based on the age of the animals and showed heterogeneous effects of high caloric diets. Our data demonstrate that particular attention needs to be paid to the timing of the diet intervention in relation to the disease process. AD progression leads to attenuation of hippocampal glucose uptake, but induction of obesity in symptomatic AD can rescue hippocampal glucose uptake while potentiating functional hyperemia. The observed benefits of HCHF diet in established AD, but not in normal aging or presymptomatic AD, are speculated to result from increased metabolite availability being propitious for the metabolically dysregulated AD brain. The advantageous effects of HCHF observed here in symptomatic AD pathology align well with human studies showing that late-life obesity is associated with a slower decline in cognitive function and reduced risk of AD progression 31. Our data indicate obesity may confer brain resilience, and thereby illuminate the changes likely underlying the obesity paradox 128.

## Supplementary Material

Supplementary information and figures.

## Figures and Tables

**Table 1:** Summary of rats used for the study by experiment, age, diet, genotype and sex (M: male; F: female)

Data/MRI protocol	Age	Diet	Total N	Genotype (sex)		attrition	reason
				nTg (M,F)	TgAD (M,F)		
**Total used in the study** **85**	6 months	CHOW	**10**	**6 (4,2)**	**4 (2,2)**		
		HCHF	**10**	**5 (2,3)**	**5 (4,1)**		
	12 months	CHOW	**22**	**12 (6,6)**	**10 (5,5)**		
		HCHF	**22**	**12 (6,6)**	**10 (5,5)**		
	15 months	CHOW	**8**	**4 (2,2)**	**4 (1,3)**		
		HCHF	**13**	**7 (3,4)**	**6 (3,3)**		
**Blood measurements**	6 months	CHOW	9	5 (3,2)	4 (2,2)	1	*b*
		HCHF	10	5 (2,3)	5 (4,1)	0	
	12 months	CHOW	21	11 (5,6)	10 (5,5)	1	*a, b*
		HCHF	22	12 (6,6)	10 (4,6)	0	
**CEST**	6 months	CHOW	10	6 (4,2)	4 (2,2)	0	
		HCHF	10	5 (2,3)	5 (4,1)	0	
	12 months	CHOW	21	11 (5,6)	10 (5,5)	1	*a, b, c, d*
		HCHF	20	11 (6,5)	9 (4,5)	2	
	15 months	CHOW	7	4 (2,2)	3 (1,2)	1	*e*
		HCHF	11	6 (2,4)	5 (2,3)	2	*f*
**pCASL* (resting state)***	6 months	CHOW	10	6 (3,3)	4 (2,2)	0	
		HCHF	7	4 (2,2)	3 (2,1)	3	*d, e*
	12 months	CHOW	22	11 (5,6)	11 (5,6)	0	
		HCHF	19	10 (5,5)	9 (3,6)	3	*d, e*
	15 months	CHOW	8	4 (2,2)	4 (1,3)	0	
		HCHF	13	7(3,4)	6 (3,3)	0	
**pCASL *(functional hyperemia)***	6 months	CHOW	10	6 (3,3)	4 (2,2)	0	
		HCHF	7	4 (2,2)	3 (2,1)	3	*d, e*
	12 months	CHOW	22	10 (4,4)	12 (3,5)	0	
		HCHF	18	10 (5,5)	8 (3,5)	4	*d, e*
	15 months	CHOW	8	4 (2,2)	4 (1,3)	0	
		HCHF	11	7 (3,4)	4 (1,3)	2	*e*

a: unsuccessful 2DG delivery b: rat expired due to surgical complications c: High B0 inhomogeneity (SD > 100) d: acquisiiton problem (set up/hardware/software) e: severe motion (motion > 1 voxel) f: did not acquire data (extra rats)
