# Supplementary material for: High caloric intake improves neuronal metabolism and functional hyperemia in a rat model of early AD pathology
Source: Theranostics. 2024 Oct 28;14(19):7405–23. doi: 10.7150/thno.98793 (PMC11626934; doi:10.7150/thno.98793)
Supplement: Supplementary file 1 — Supplementary information and figures. [file thnov14p7405s1.pdf]

SUPPLEMENTARY TEXT

HCHF-fed rats gain weight

Notably, the increase in total calories consumed was due to the consumption of the 12% sucrose solution: after removing the calories consumed through its drinking, the average calories consumed by HCHF-fed rats were comparable with the average calories consumed by rats fed with only CHOW (***Figure 2.A.iii***). Compared to rats on CHOW only, HCHF-fed rats consumed more fats (6 months: 127 ± 29 kcal/week, $P = 0.033$; 12 months: 111 ± 27 kcal/week, $P = 0.043$) (***Figure 2.A.ii***), and more carbohydrates (6 months: 165 ± 32 kcal/week, $P = 0.009$; 12 months: 169 ± 29 kcal/week, $P = 0.004$) (***Figure 2.A.iii***).

SUPPLEMENTARY FIGURES

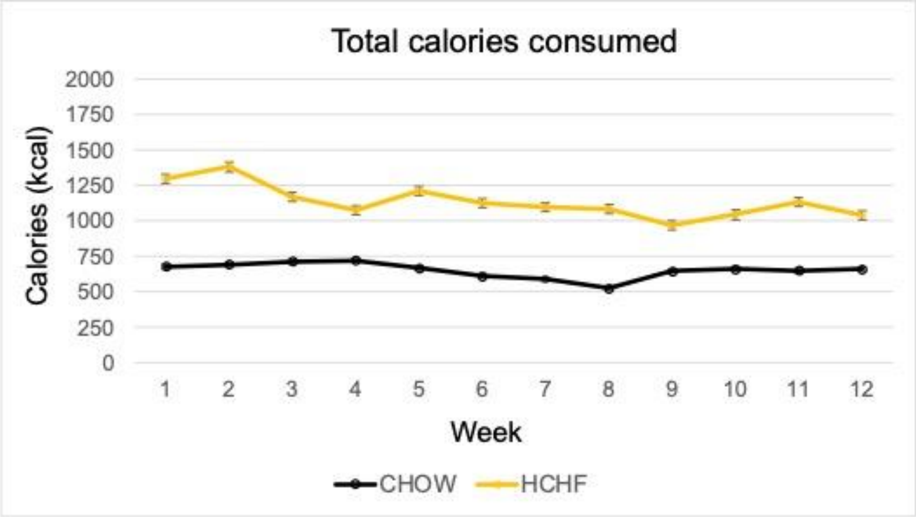

Figure S1: **Average total calories consumed per rat cage per week.** HCHF-fed rats had consistently higher total average calories consumed per week compared to CHOW-fed rats.

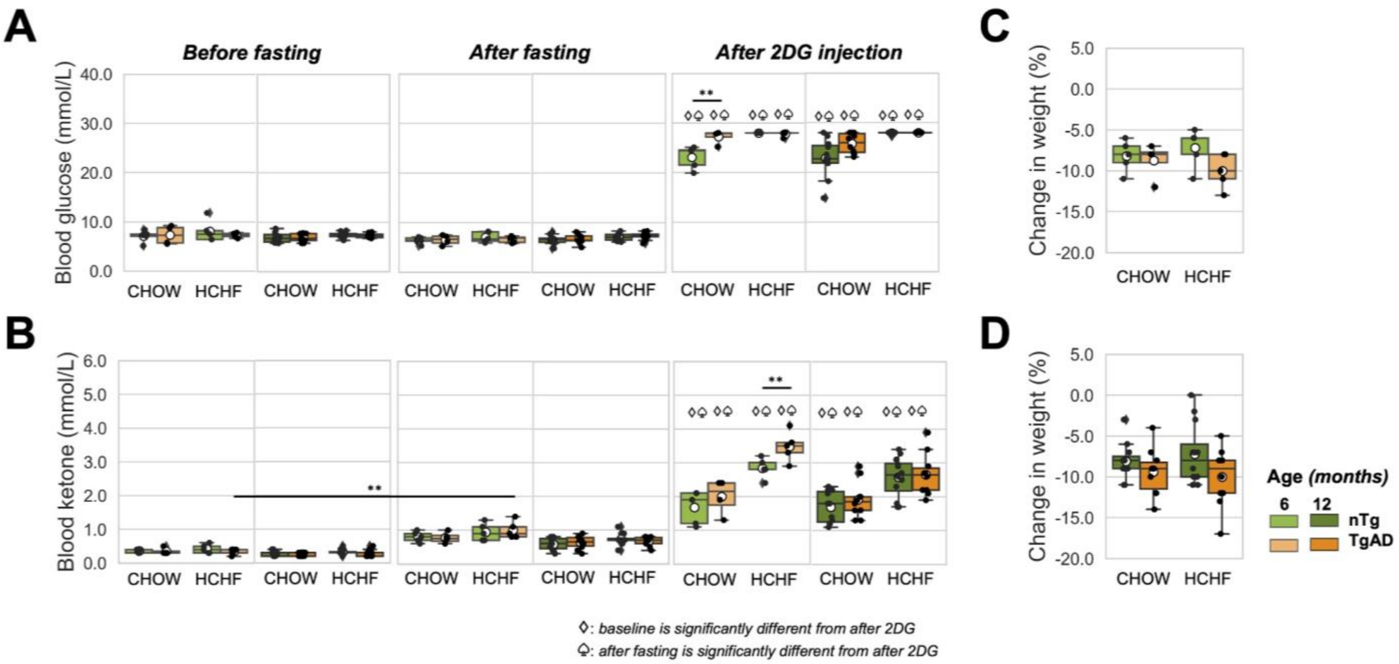

Figure S2: **2DG administration results in higher blood glucose and BHB in HCHF-fed cohorts when compared to CHOW-fed cohorts**. **(A)** Baseline blood glucose levels at 6 months of age were indistinguishable. At 12 months of age, the HCHF-fed cohort has higher baseline (by 1 ± 1 mmol/L, $P < 0.001$) blood glucose when compared to that of the CHOW-fed cohort. Blood glucose levels after fasting were indistinguishable at 12 months of age. At 6 months of age, blood glucose after fasting was higher in the nTg vs. in the TgAD cohort (by 0.4 ± 0.2 mmol/L, $P = 0.04$). After 2DG injection, CHOW-fed nTg rats had lower blood glucose (by -5.0 ± 0.6 mmol/L, $P < 0.001$ at 6 months; by -5.1 ± 0.6 mmol/L, $P < 0.001$ at 12 months) compared to HCHF-fed nTg rats and (by -4.2 ± 0.6 mmol/L, $P = 0.002$ at 6 months; by -3.0 ± 0.7 mmol/L, P = 0.018 at 12 months) when compared to CHOW-fed TgAD rats. **(B)** Baseline blood BHB levels of 6-month-old nTg rats were higher (by 0.08 ± 0.02 mmol/L, $P < 0.001$) compared to TgAD rats. After fasting, blood BHB of HCHF-fed 6-month-old rats were higher (by 0.23 ± 0.07 mmol/L, $P = 0.002$) compared to CHOW-fed rats. After 2DG injection, HCHF-fed cohorts had higher blood BHB (by 1.5 ± 0.3 mmol/L, P < 0.001 at 6 months; 0.8 ± 0.2 mmol/L, *P* <

*0.00*1 at 12 months) compared to CHOW-fed cohorts. **(C)** TgAD rats showed greater decrease in weight after overnight fasting compared to nTg rats **(C)** at 6 months (by -3 ± 1%, $P = 0.004$) and **(D)** at 12 months (by -3 ± 1%, $P = 0.013$). **(D)** 12-month-old males showed greater decrease in weight (by -4 ± 1%, $P = 0.008$) after overnight fasting than the females. (Note: limit of detection of the glucometer used is 28 mmol/L.). *$P < 0.05$, **$P < 0.01$.

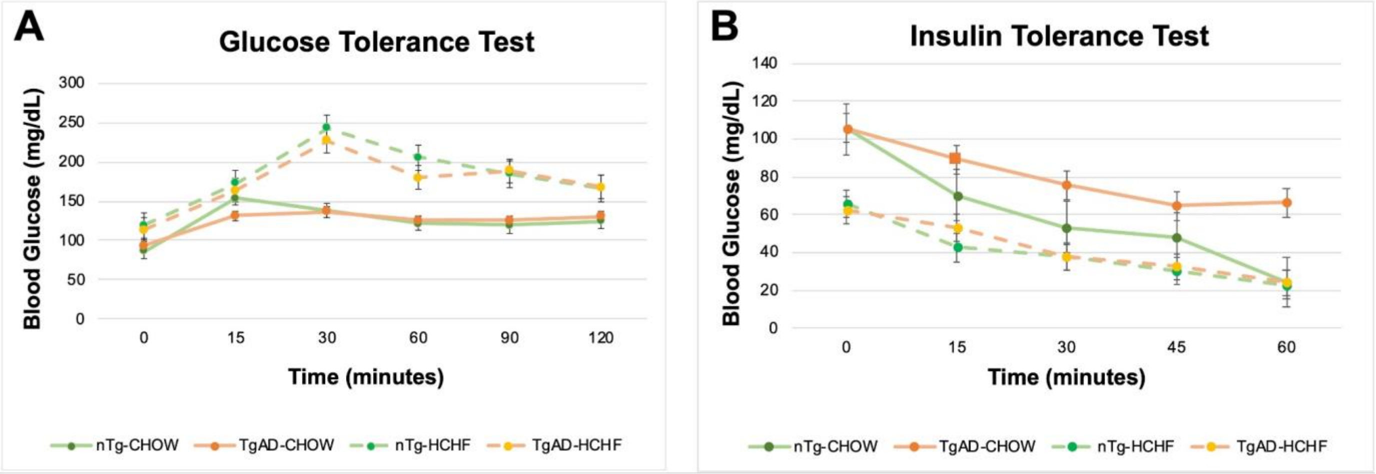

***Figure S3*: Blood glucose measurement during glucose tolerance and insulin tolerance tests.** A benchtop experiment was conducted to assess glucose and insulin tolerance of the F344 rats. For the glucose tolerance test (GTT), rats were fasted for 12 hours beginning at the time of lights-on (8pm-8am). Blood sample was collected before oral administration of glucose (time “0” minutes). Rats were anesthetized lightly (~3% isoflurane for 1.5 minutes) via nose cone to gavage glucose (2g/kg concentration of 40% D-glucose in saline). **(A)** Blood glucose was then assessed at 15, 30, 60, 90, and 120 minutes following glucose administration. The blood glucose levels at 120 minutes were not statistically significantly different from their corresponding levels at time “0” in HCHF-fed rats ($P = 0.070$) but they were elevated, relative to baseline, in CHOW-fed rats ($P < 0.001$). When compared to CHOW-fed rats, HCHF-fed animals had higher fasting blood glucose at post-fasting time “0”, prior to D-glucose administration (diet, $P < 0.001$), likely due to excess carbohydrates of the HCHF diet. Furthermore, per time point analysis showed elevated blood glucose of HCHF-fed rats compared to those of CHOW-fed rats at all time points (diet effect | 15 mins, $P = 0.001$; 30 mins, $P < 0.001$; 60 mins, $P = 0.001$; 90 mins, $P < 0.001$; 120 mins, $P < 0.001$), suggesting that the glucose clearing in HCHF-fed rats is altered, relative to that of CHOW-fed rats. For the insulin tolerance test (ITT), rats were fasted for 6 hours beginning at the time of lights-off (6:30am-12:30pm). **(B)** Blood sample was collected to evaluate blood glucose level before IP administration of human insulin (time “0” minutes). Blood glucose was then assessed at 15, 30, 45, and 60 minutes after IP injection of 1 IU/kg dose of human insulin. Linear mixed effects modeling of the ITT data revealed that HCHF-fed rats had lower fasting blood glucose at post-fasting time “0” (diet, $P < 0.001$), indicating a greater insulin response to fasting in HCHF-fed rats at the onset of their “active phase”. Evaluating each group, CHOW-fed nTg rats exhibited significant blood glucose concentration clearance, relative to post-fasting glucose, at all time points (15 mins, $P < 0.001$; 30 mins, $P < 0.001$; 45 mins, $P < 0.001$; 60 mins, $P < 0.001$). CHOW-fed TgAD rats showed blood glucose clearance beginning at 30 mins (30 mins, $P = 0.014$; 45 mins, $P = 0.001$; 60 mins, $P = 0.01$). In HCHF-fed rats, both nTg (15 mins, $P = 0.007$; 30 mins, $P = 0.001$; 45 mins, $P < 0.001$; 60 mins, $P < 0.01$) and TgAD (15 mins, $P = 0.042$; 30 mins, $P < 0.001$; 40 mins, $P < 0.001$; 60 mins, $P < 0.001$) cohorts also showed significant blood glucose clearance, relative to fasting. Therefore, within each diet, the glucose response to insulin demonstrated the expected time-dependent decrease [48]. To assess insulin resistance in each group, the slope of the line of best fit was calculated. The CHOW-fed nTg rats exhibited the steepest slope (-18.5 mg/dL/min), indicating efficient glucose clearance, suggesting a non-diabetic state. In contrast, CHOW-fed TgAD rats (-10.3 mg/dL/min), as well as both HCHF-fed nTg (-9.8 mg/dL/min) and TgAD rats (-9.8 mg/dL/min), displayed significantly slower glucose clearance. This slower response points

to reduced insulin sensitivity, potentially indicative of early onset of insulin resistance in these groups. Overall, the decline of blood glucose after insulin injection - signifying insulin regulation and response - remains competent in both nTg and TgAD rats regardless of diet. A total of N=8 rats were used in GTT and ITT, where n=4 (2 nTg, 2 TgAD) were fed with CHOW and n=4 (2 nTg, 2 TgAD) were fed with HCHF.

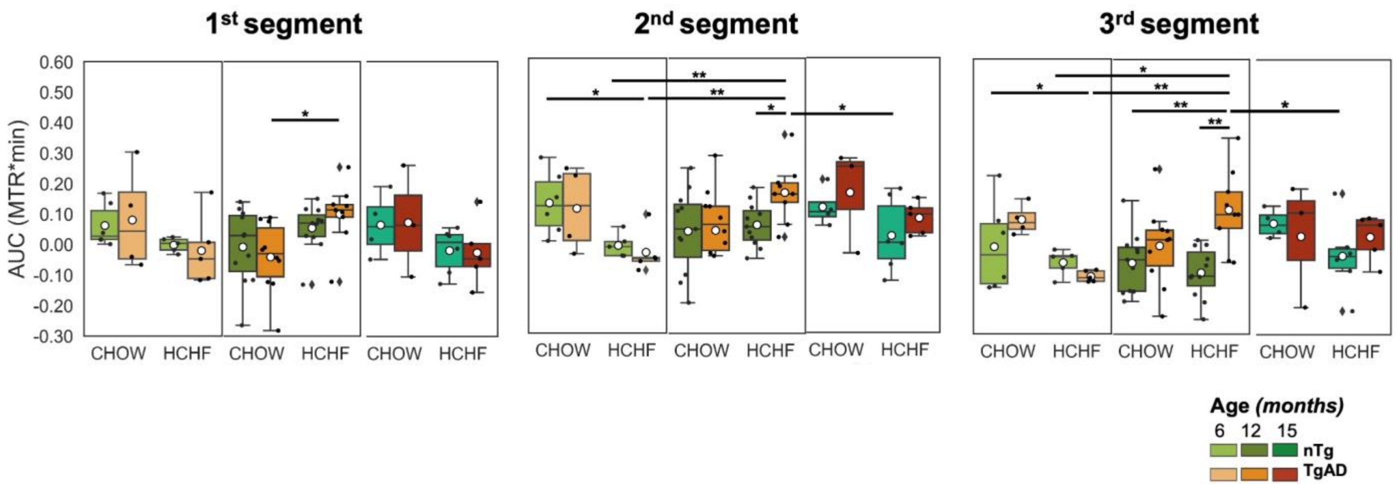

***Figure S4*: Hippocampal glucose uptake per 20-min segment**. At 6 months, HCHF-fed rats had lower AUC at initial uptake and peak uptake phases when compared to CHOW-fed rats (1st segment: diet, $P < 0.01$; 2nd segment: diet, $P < 0.001$). There was a significant interaction of age and genotype in glucose uptake in the peak (1st segment: $P < 0.01$) and washout phases (3rd segment, $P < 0.001$) of HCHF-fed rats. Where 12-month-old HCHF-fed TgAD rats (peak phase: 0.2 ± 0.1; washout phase: 0.1 ± 0.1) had greater AUC when compared to 12-month-old HCHF-fed nTg (peak phase: 0.06 ± 0.07; washout phase: -0.10 ± 0.08), 6-month-old HCHF-fed nTg (peak phase: -0.01 ± 0.04; washout phase: -0.07 ± 0.04), TgAD rats (peak phase: -0.04 ± 0.07; washout phase: -0.11 ± 0.02) and 15-month-old HCHF-fed nTg rats (peak phase: 0.02 ± 0.12; washout phase: -0.04 ± 0.13). *$P < 0.05$, **$P < 0.01$.

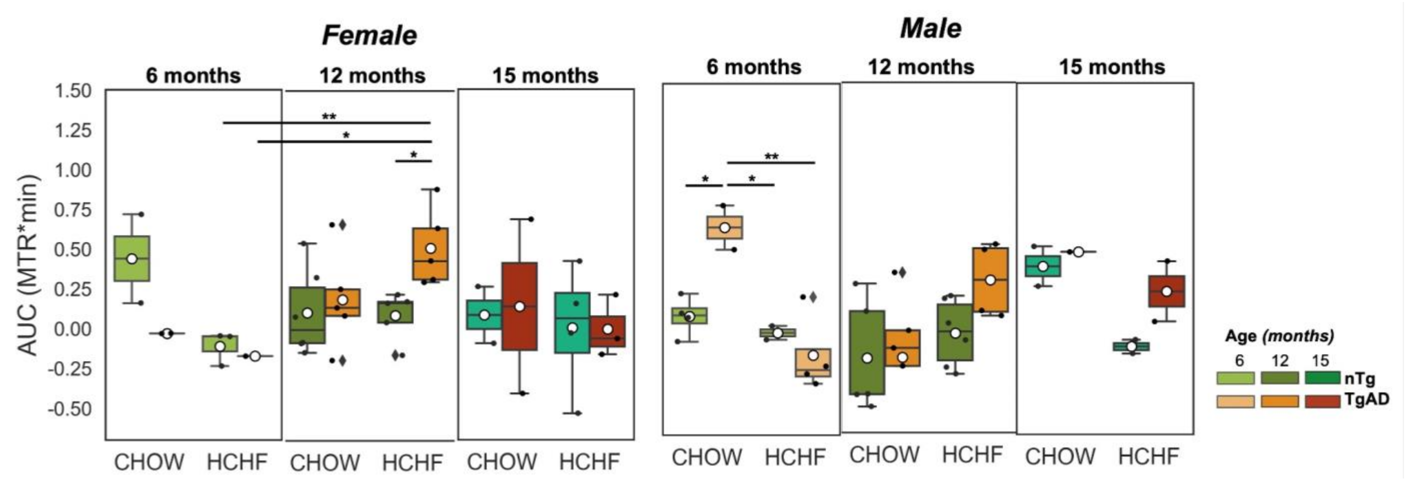

***Figure S5*: Sex difference in hippocampal glucose uptake**. AUC showed sex differences over 60 minutes after the onset of 2DG infusion for 6- and 12--month-old cohorts (6 months: diet-genotype-sex, $P < 0.001$; 12 months: genotype-sex, $P < 0.01$). At 6 months of age, HCHF-fed rats had lower AUC compared to CHOW-fed rats (diet, $P < 0.001$). However, considering 6-month-old male rats, CHOW-fed TgAD rats (0.5 ± 0.1) had higher AUC compared to CHOW-fed nTg rats (-0.04 ± 0.51) and HCHF-fed nTg (-0.12 ± 0.04) and TgAD rats (-0.12 ± 0.25). CHOW-fed cohorts at 6 months, female nTg rats had a trend of higher glucose uptake compared to male nTg rats while male TgAD rats had a trend of higher glucose uptake compared to female TgAD rats. At 12 months, there was a significant interaction of diet and genotype ($P < 0.001$) where HCHF-fed female rats

(0.5 ± 0.3) had larger AUC compared to HCHF-fed female nTg (0.03 ±) and to 6-month-old HCHF-fed female nTg (-0.08 ± 0.09) and TgAD rats (-0.03). *$P < 0.05$, **$P < 0.01$.

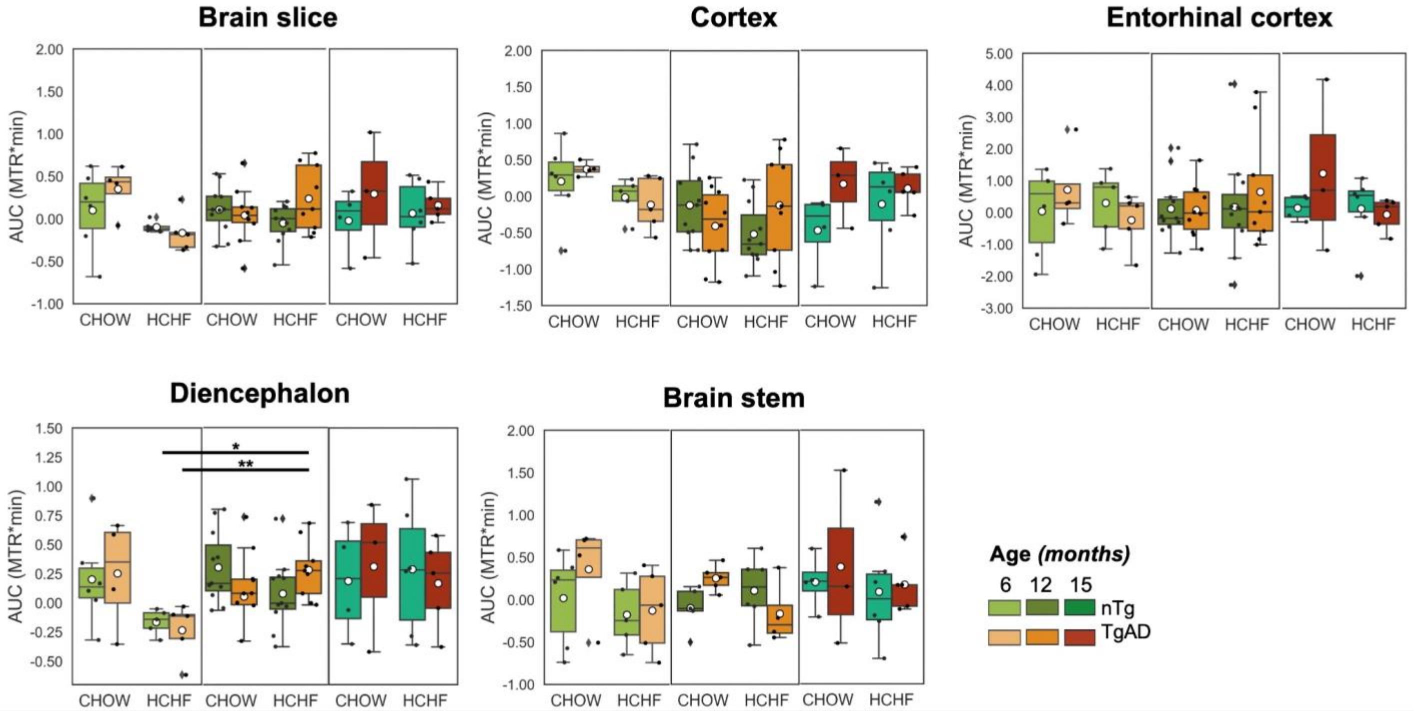

Figure S6**: Hippocampal glucose uptake in different regions of interests**. The interaction of diet and genotype at 12 months observed in the hippocampus was also observed in the brain slice (*$P < 0.01$*), cortex (*$P < 0.01$*) and diencephalon (*$P < 0.05$*). However, after pairwise comparison, only the 12-month-old HCHF-fed

TgAD rats (0.3 ± 0.3) in diencephalon demonstrated larger AUC compared to 6-month-old HCHF-fed nTg (-0.2 ± 0.1) and TgAD (-0.2 ± 0.2) rats. *$P < 0.05$, **$P < 0.01$.

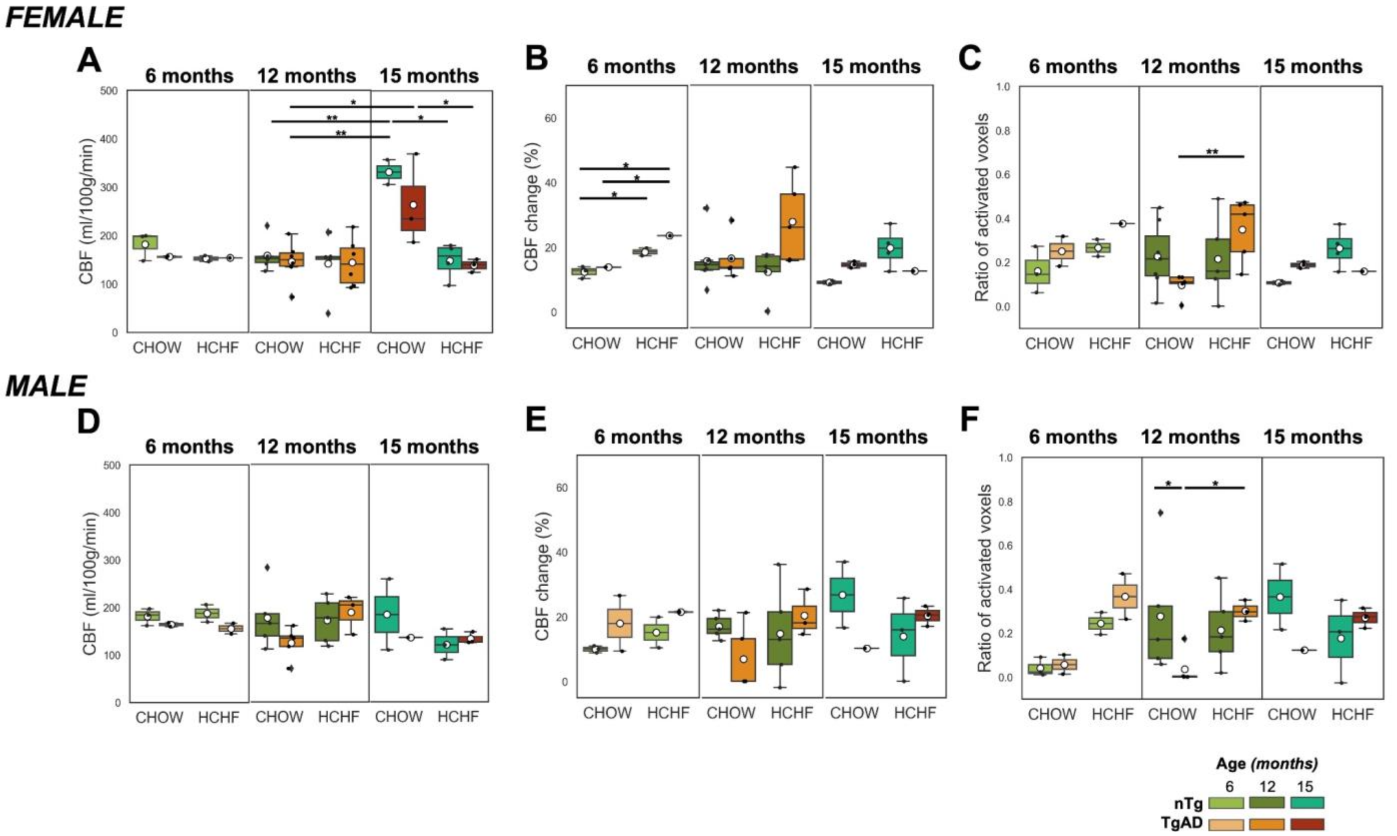

***Figure S7*: Sex difference in resting CBF and functional hyperemia**. There was a significant sex effect in resting CBF at 6 (diet-sex, $P < 0.05$; sex, $P < 0.01$) and 15 months (diet-sex, $P < 0.05$). At 6 months, HCHF-fed female rats had a trend of lower resting CBF compared to male rats. However, at 15 months, CHOW-fed female rats had higher resting CBF compared to male rats. There was no significant sex effects in CBF change and area of activation in response to forepaw stimulation at 6 and 12 months. With prolonged HCHF diet at 15 months, there was only a significant sex effect in CBF change in response to stimulation despite having low number (diet-genotype-sex, $P < 0.05$; diet-sex, $P < 0.05$) where female CHOW-fed nTg rats had a trend of lower CBF change compared to male CHOW-fed nTg rats. In addition, female HCHF-fed nTg rats had a trend

of higher CBF response compared to female HCHF-fed TgAD rats and the trend was reversed in male rats. *$P < 0.05$, **$P < 0.01$.

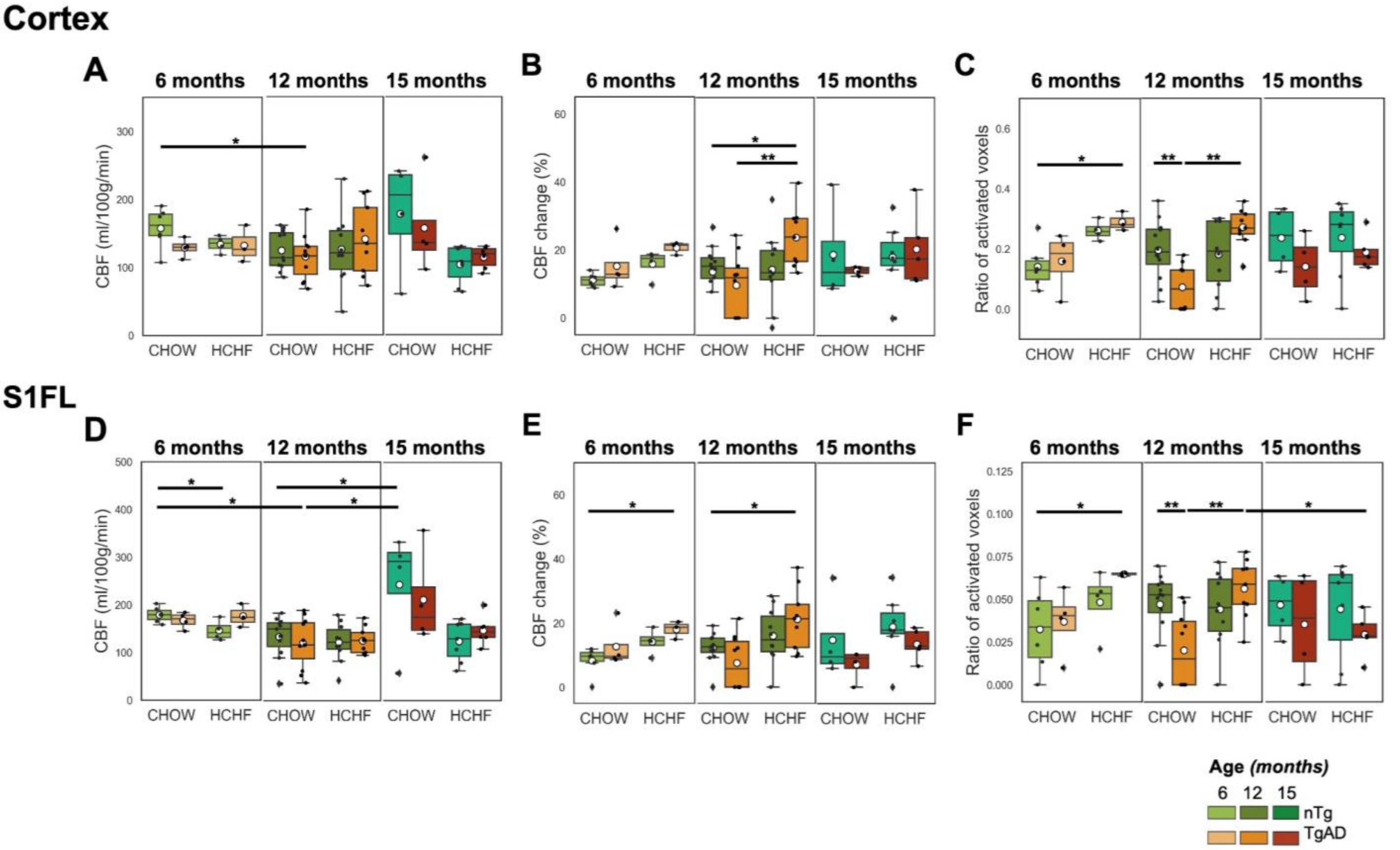

***Figure S8*: Resting CBF and functional hyperemia in different regions of interests.** The observed contrasts in resting CBF and functional hyperemia in the brain slice were also demonstrated in cortex and in the primary somatosensory area for the forelimb (S1FL). At 6 months, diet effect was only observed in resting CBF in S1FL (diet, $P < 0.001$) while area of activation in response to forepaw stimulation was only observed in cortes (diet, $P < 0.001$). However, in CBF change in response to forepaw stimulation, diet effect was observed in both cortex ($P < 0.001$) and S1FL ($P < 0.01$). At 12 months, both cortex (CBF change: diet-genotype, $P < 0.05$; area of activation: diet-genotype, $P < 0.001$) and S1FL (CBF change: diet-genotype, $P < 0.05$; area of activation: diet-genotype, $P < 0.01$) demonstrated an interaction of diet and genotype in functional hyperemia. At 15 months,

all regions showed that CHOW-fed rats had higher resting CBF compared to HCHF-fed rats (cortex: diet, $P < 0.05$; S1FL: diet, $P < 0.01$) and no differences in functional hyperemia. *$P < 0.05$, **$P < 0.01$.

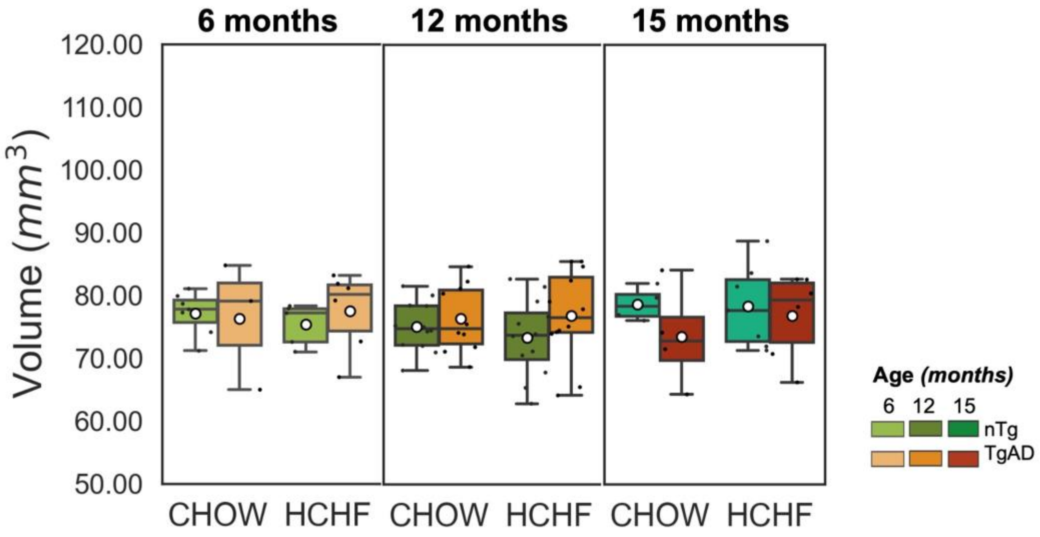

***Figure S9*: Hippocampal volume across all groups.** Estimated hippocampal volumes showed no significant differences across age groups, genotypes, or diet exposure. At 6 months of age, n=21, where n=10 (7 nTg, 3 TgAD) are CHOW-fed and n=11 (5 nTg, 6 TgAD) are HCHF-fed. At 12 months of age, n=46, where n=22 (12 nTg, 10 TgAD) are CHOW-fed and n=24 (12 nTg, 12 TgAD) are HCHF-fed. At 15 months of age, n=21, where n=8 (4 nTg, 4 TgAD) are CHOW-fed and n=11 (7 nTg, 6 TgAD) are HCHF-fed.
